# A systematic review of *Moringa oleifera* seed toxicity and safety: Comparative analysis of toxicology methodologies and results

**DOI:** 10.1371/journal.pone.0360006

**Published:** 2026-11-06

**Authors:** Joseph James Russell, Martin Pritchard, Andrew W. J. Paterson

**Affiliations:** 1 School of Built Environment, Engineering and Computing, Leeds Beckett University, Leeds, United Kingdom; 2 School of Health, Leeds Beckett University, Leeds, United Kingdom; Institute for Biological Research, University of Belgrade, SERBIA

## Abstract

This systematic review aims to assess the reliability of contemporary toxicological research on *Moringa oleifera* seeds. Academic databases were browsed on a yearly basis (2014–2024); identification and screening were conducted between July 17^th^ 2023 and January 13^th^ 2025. Primary research reports on *M. oleifera* seed toxicology were included for data collection. Fifty-three fields were used to collect data. Data was synthesised by comparing methodological data points to contemporary toxicity testing guidelines; results were presented as a narrative synthesis. Conflict of interest, research quality and risk of bias were assessed using Cochrane tools modified for non-human laboratory studies. Thirty-seven non-duplicate publications were identified, of which 14 were included for data collection. In total, 395 data points were collected and synthesised. Financial conflicts of interest were declared in one report. Nine reports received “lower quality” or “lowest quality” research quality judgements. Six reports received “high risk of bias” judgements. Bias risk was commonly associated with selective reporting of data. *M. oleifera* seed toxicity was commonly tested via rodent study. Seven rodent studies included an acute toxicity test and nine included a sub-acute toxicity test. Two acute oral toxicity tests returned LD50 values of 14,000–15,000 mg/kg bw. Three sub-acute oral toxicity tests returned NOAEL values of 100–257 mg/kg bw/d. Oral LD50 and NOAEL data could only be derived from reports with “lowest quality” research and/or “high risk of bias” judgements, so these safety limits are unreliable. Inadequate dosing schemes were the primary cause of limited LD50 and NOAEL data. Dose range non-compliance also caused unnecessary animal suffering. To facilitate standardisation in future research, several guideline-based methodological recommendations are made. Determining reliable safety limits for *M. oleifera* seed consumption may reduce incidence of acute kidney injury, thereby supporting Targets 3.4 and 3.d of the United Nations sustainable development goals.

## 1. Introduction

Acute kidney injury (AKI) is disproportionately common in sub-Saharan Africa. Incidence of AKI in sub-Saharan Africa can range from 5.7–22.3% in general hospital admissions and can exceed 50% in emergency admissions [1]. For comparison, incidence of AKI in the United Kingdom is 5.4–7.6% in general hospital admissions and approximately 20% in emergency admissions [2–4]. AKI in sub-Saharan Africa is associated with costly treatment, prolonged hospitalisation and high in-hospital mortality rates, which can range from 37–58% [1]; therefore, the disease burden is high.

Infectious disease is the leading cause of AKI in sub-Saharan Africa, with sepsis present in 26–50% of cases [1]. Nephrotoxic agents, obstetrical complications, perioperative complications and obstructive uropathy are also significant causes and contributing factors of AKI [1]. Nephrotoxicity is commonly attributed to nephrotoxic medications, but medicinal herbs can also be a common cause [5]. In a 2018 study, Halle *et al.* [6] reported herbal intoxication as the leading cause of nephrotoxic AKI cases in a Cameroonian hospital, at 32.3%. Minja *et al.* [7] suggest herbal intoxication is more prevalent in low-resource settings and more commonly affects young people than other causes of AKI. Masewu *et al.* [5] suggest medicinal herbs are unsafe because toxicological investigations—which should be used to determine safety limits—are not being performed systematically.

An alternate perspective elucidates a 5000-year history of medicinal plant usage. Petrovska (2012) states “*The continuous and perpetual people's interest in medicinal plants has brought about today's modern and sophisticated fashion of their processing and usage*” [8]. Researchers who subscribe to this notion often apply a “wisdom of the ages” or “folkloric” approach; that is to suggest, sustained historical use of plant products is evidence of efficacy and safety in medicinal and nutritional contexts. Petrovska (2012) goes on to state “*Contemporary science has acknowledged the active action [of medicinal plants], and it has included in modern pharmacotherapy a range of drugs of plant origin, known by ancient civilizations and used throughout the millennia*” [8]. This is particularly true of Chinese herbal medicine, the study of which can be traced to the Eastern Han Dynasty (25–220 AD). The earliest extant pharmacopoeia, the *Shen Nong Ben Cao Jing*, grades plant products on their medicinal efficacy, toxicity and compatibility with other substances [9]. Therefore, toxicology has been a core element of pharmacognosy for at least 2000 years, indicating ancient peoples concerned themselves with both efficacy and safety; as should modern researchers.

*Moringa oleifera* Lamarck (MO)—commonly known as the “drumstick tree”, “ben oil tree”, “miracle tree” or “tree of life”—is indigenous to Northern India and naturalised in tropical and subtropical regions of North America, South America, Africa, Asia and Australia [10]. The propagation of MO can largely be attributed to the nutritional profile of its leaves and seed pods, the oil content of its seeds and medicinal properties reported in all parts of the plant. MO seeds can also be used as a natural coagulant for treatment of drinking water in low-resource settings. The adoption of MO as a drought-resistant source of food and medicine has been rapid, whereas its adoption as a natural coagulant is still experimental. Over the past decade, interest in MO research has grown in response to the tree’s rapid adoption and folkloric reputation. MO leaves and seeds are the most commonly studied plant materials, whereas the edible seed pods are rarely studied. In response to the “silent pandemic” of kidney disease, many researchers engage in plant bioprospecting to discover and develop new nephroprotective compounds. A common aim of this research is to prevent clinically-acquired AKI by developing nephroprotective compounds to counteract nephrotoxic medicines. To date, research on the nephroprotective effects of MO seed are of this nature, as demonstrated by two 2019 publications that report nephroprotection versus gentamicin [11,12]; a common cause of AKI [13]. To assess the utility of MO seed as a nephroprotective agent, the material must first be studied in a toxicological context to ensure safety. If MO seed is toxic, it may be unsuitable for clinical use and act as a confounding factor in drug discovery trials of this type.

The authors of this review are currently investigating the viability of MO seed as an alternative to chemical coagulants for treatment of drinking water in low-resource settings; safety is the foremost priority of this investigation. There is a mixed-messaging regarding MO seed safety in early toxicology research (2016–2017), wherein individual reports suggest both caution and safe consumption [14–16]. More recent reports (2018–2023) suggest only caution, and Ezemagu *et al.* [17] suggest there is an urgent need to discourage MO seed consumption and standardise investigations into MO seed water treatment [18,19]. To date, there are no internationally-recognized safety limits for consumption or medical usage of MO seeds. As such, the food safety authorities of multiple nations have prohibited Moringa products: As of 2022, the European Union “*does not allow the use of moringa in herbal medicinal products or the use of moringa seed oil in food supplements*” [20]; As of 2025, Brazil’s ANVISA continues to prohibited the manufacture, import, sale, advertising and distribution of all foods containing MO, citing limited safety evidence and spurious therapeutic claims [21]; As of 2025, the Food Standards authority of Australia and New Zealand (FSANZ) does not permit MO “*as a food or food ingredient for retail sale*”, citing insufficient evidence to establish safety for human consumption [22]. For MO seed water treatment to progress beyond the experimental stage, researchers must determine oral toxicological safety limits for MO seed. Research must be consistent and of a high standard to facilitate safe integration of the technology.

This review aims to assess the reliability of contemporary research (2014–2024) on MO seed toxicology, with emphases on methodological consistency, standardisation and animal welfare. An additional aim is to extract and compare safety limits from the included studies, and to determine whether safety limits are reliable enough to inform policy: Assessments for conflict of interest, risk of bias and research quality are employed to achieve this aim. Toxicological findings are inspected for data relevant to AKI, to determine whether MO seeds should be considered a nephrotoxic herbal product. If MO seed is consistently and reliably found to be non-toxic, the reported nutritional properties may cement the species’ status as a drought-resistant food source. This could aid the United Nations (UN) in achieving Sustainable Development Goal 2 (“*End hunger, achieve food security and improved nutrition and promote sustainable agriculture*”) [23]. If MO seeds are found to be toxic, and toxicity can be managed using reliable safety limits, the findings of this systematic review may contribute to Sustainable Development Goal 3.4 (“b*y 2030, reduce by one third premature mortality from non-communicable diseases through prevention and treatment and promote mental health and well-being*”) and goal 3.d (“*strengthen the capacity of all countries, in particular developing countries, for early warning, risk reduction and management of national and global health risks*”) [24]. In additional to achieving the aforementioned aims, this systematic review makes guideline-based recommendations for high-quality toxicological research, highlights emerging trends in contemporary research and suggests several avenues for future research.

## 2. Methodology of the systematic review

Academic databases and search engines (ScienceDirect, ResearchGate and Google Scholar) were selected for global accessibility and browsed using search terms (n = 27) created from combinations of prefix (n = 3) and suffix (n = 9) terms, as shown in Table 1.

**Table 1:** Matrix of prefix and suffix terms combined to create search terms.

	Prefix terms (n = 3)		
Suffix terms (n = 9)	Moringa	*Moringa oleifera*	*Moringa oleifera* seed
**safety**	Moringa safety	*Moringa oleifera* safety	*Moringa oleifera* seed safety
**toxic**	Moringa toxic	*Moringa oleifera* toxic	*Moringa oleifera* seed toxic
**toxicity**	Moringa toxicity	*Moringa oleifera* toxicity	*Moringa oleifera* seed toxicity
**toxicology**	Moringa toxicology	*Moringa oleifera* toxicology	*Moringa oleifera* seed toxicology
**toxicological**	Moringa toxicological	*Moringa oleifera* toxicological	*Moringa oleifera* seed toxicological
**cytotoxic**	Moringa cytotoxic	*Moringa oleifera* cytotoxic	*Moringa oleifera* seed cytotoxic
**cytotoxicity**	Moringa cytotoxicity	*Moringa oleifera* cytotoxicity	*Moringa oleifera* seed cytotoxicity
**histology**	Moringa histology	*Moringa oleifera* histology	*Moringa oleifera* seed histology
**histological**	Moringa histological	*Moringa oleifera* histological	*Moringa oleifera* seed histological

Search filters were used to browse results on a year-by-year basis, from 2014 to 2024. For each search term within each year, the first twenty results were browsed for relevant publications. Publications with primary research into MO seed toxicology were deemed relevant and included; no limitation on studied type (in vivo, in vitro, etc.) was applied. Reviews, reports containing secondary data and reports containing irrelevant primary data were excluded from data collection. Toll access reports were also excluded to improve accessibility in developing regions.

Collected data were entered into a spreadsheet with 53 data fields. All data fields concerned the methodology or results of the included reports, as shown in Table 2. Data conversions were used to standardise units of measurement for ease of comparison within each data field (e.g., g/kg bw to mg/kg bw, °F to °C / K).

**Table 2:** Fields used to collect data from publications included in the systematic review.

Data field
Source(s) of MO seeds
Method of species verification
Age of MO seeds at time of testing
Storage conditions of MO seeds
MO seed primary processing methods
MO seed defatting methods
MO seed secondary processing methods
MO seed tertiary processing methods
Maximum processing temperature
Final MO seed dose type(s)
Species/breed of test organism(s)
Age of rodents at time of testing
Rodent acclimatisation duration
Rodent housing temperature
Rodent housing humidity
Rodent housing light cycle
Rodent diet during acclimatisation
Rodents per cage
Adherence to ethical guideline(s) for animal testing
Rodent fasting practices
Method(s) of MO seed administration
Dose administration vehicle
Acute toxicology: Total number of test organisms
Acute toxicology: Test organisms per group
Acute toxicology: Number of dosed groups
Acute toxicology: Inclusion of negative control group (Y/N)
Acute toxicology: MO seed dosing concentration(s)
Acute toxicology: Duration of observations
Acute toxicology: Method(s) of data/statistical analysis
Sub-acute toxicology: Total number of test organisms
Sub-acute toxicology: Test organisms per group
Sub-acute toxicology: Number of dosed groups
Sub-acute toxicology: Inclusion of negative control group (Y/N)
Sub-acute toxicology: Acute toxicology: MO seed dosing concentration(s)
Sub-acute toxicology: Duration of study
Sub-acute toxicology: Method(s) of data/statistical analysis
Acute toxicology: Mortality rate
Acute toxicology: LD50
Acute toxicology: Clinical observations
Acute toxicology: Additional observations
Sub-acute toxicology: NOAEL
Sub-acute toxicology: Mortality rate
Sub-acute toxicology: Clinical observations
Sub-acute toxicology: Food consumption rate change
Sub-acute toxicology: Body weight change
Sub-acute toxicology: Changes to sensory reactivity
Sub-acute toxicology: Haematological changes
Sub-acute toxicology: Changes to biochemistry: Renal panel
Sub-acute toxicology: Changes to biochemistry: Hepatic panel
Sub-acute toxicology: Gross necropsy observations
Sub-acute toxicology: Histopathological changes
Sub-acute toxicology: Reproductive pathology
Sub-acute toxicology: Additional observations
**Total data fields: 53**

Methodological data were synthesised in comparison to OECD guidelines for oral toxicity testing. Each data point was inspected for compliancy with corresponding guideline values in relevant OECD methodologies. Compliancy rates were then used to assess the quality and consistency of the included reports.

Each report was simultaneously assessed for conflict of interest, research quality and risk of bias. Conflict of interest assessments were conducted by examining conflict of interest statements, funding statements and the research institutes affiliated with each contributing author. A conflict of interest judgement (CJ) was then made for each report using the criteria in Table 3. This methodology was taken directly from the Cochrane Handbook for Systematic Reviews [25].

**Table 3:** Criteria used to determine the conflict of interest judgement (CJ).

Conflict of interest judgement (CJ)	Judgement criteria
No notable concern about conflict of interest	No declared conflict of interest and no apparent undeclared conflict of interest.
Notable concern about conflict of interest	Declared conflict of interest and/or apparent undeclared conflict of interest.

Research quality assessments were conducted using ten common signalling questions, as shown in Table 4. All signalling questions were phrased to produce one of three judgements: “yes” (Y) indicated a positive outcome, “unclear” (U) indicated uncertainty and “no” (N) indicated a negative outcome. Reasoning was recorded for all “unclear” and “no” judgements. A quality score (QS) was assigned to each report using the following system: each “yes” judgement returned two points, each “unclear” judgement returned one point and all “no” judgements returned zero points. As such, the maximum QS was twenty points. An overall quality judgement (QJ) was then made for each report using the criteria in Table 5. This system was adapted from signalling questions listed by Al-Jundi and Sakka (2017) [26].

**Table 4:** Signalling questions used to assess research quality.

Code	Quality assessment signalling question
Q-Q1	Are procedures fully described?
Q-Q2	Are procedures appropriate?
Q-Q3	Is the group size/number of replicates appropriate?
Q-Q4	Do procedures include appropriate control groups?
Q-Q5	Are procedures applied identically to all groups?
Q-Q6	Are procedures replicable?
Q-Q7	Is analysis fully described?
Q-Q8	Is analysis appropriate?
Q-Q9	Is analysis applied identically to all groups?
Q-Q10	Are data fully reported?

**Table 5:** Criteria used to determine the overall research quality judgement (QJ).

Overall research quality judgement (QJ)	Judgement criteria
Highest quality	Quality score (QS): 19/20–20/20
Higher quality	Quality score (QS): 17/20–18/20
Lower quality	Quality score (QS): 15/20–16/20
Lowest quality	Quality score (QS): 0/20–14/20

Risk of bias assessments were conducted using ten common signalling questions under three bias domains, as shown in Table 6. All signalling questions were phrased to produce one of three answers: “yes”, “unclear” or “no”. Unlike the research quality assessment, answers to signalling questions were not indicative of positive, uncertain or negative outcomes. Answers to signalling questions were instead passed through one of three flowcharts dedicated to each bias domain (Section 7 – Supporting information: S1 Fig, S2 Fig and S3 Fig). Risk of bias judgements for each bias domain were reached by following these flowcharts to their conclusions. An overall risk of bias judgement (B-J4) was then made for each report using the criteria in Table 7. This methodology was adapted from the Cochrane Handbook for Systematic Reviews and Cochrane ROBINS-E tool, to function with non-human laboratory studies [27,28].

**Table 6:** Bias domains and signalling questions used to assess risk of bias.

Code	Bias domain / Risk of bias signalling question
**B-J1**	**Bias domain 1:** Risk of bias due to experimental design and confounding factors
B-Q1.1	Does the design of the experiment preclude bias?
B-Q1.2	Are confounding factors identified, measured, controlled and reported?
B-Q1.3	Do negative control data suggest confounding?
**B-J2**	**Bias domain 2:** Risk of bias due to missing data
B-Q2.1	Are results data fully reported?
B-Q2.2	Are data pertaining to confounding factors fully reported?
B-Q2.3	Are missing data likely to affect the perception of *M. oleifera* seed toxicity?
**B-J3**	**Bias domain 3:** Risk of bias due to selective reporting of data
B-Q3.1a	Were data collected, analysed or presented using multiple methods in parallel?
B-Q3.1b	Do conclusions consider data generated by all parallel methods?
B-Q3.2	Are conclusions drawn from desirable data from a select sub-group or sub-groups?
B-Q3.3	Are conclusions made in omission of undesirable data from a select sub-group or sub-groups?

**Table 7:** Criteria used to determine overall risk of bias (B-J4).

Overall risk of bias judgement (B-J4)	Judgement criteria
Low risk of bias	The study was judged to have low risk of bias in all bias domains.
Some concerns	The study raised some concerns in one or two bias domains, but was not judged to have high risk of bias in any bias domain.
High risk of bias	The study was judged to have high risk of bias in at least one bias domain or some concerns in all bias domains.

The identification and screening processes occurred between July 17^th^ 2023 and January 13^th^ 2025 and data collection and synthesis occurred between July 31^st^ 2023 and January 17^th^ 2025. Identification, screening, data collection and synthesis were conducted in duplicate using no automation tools. Assessments for conflict of interest, research quality and risk of bias were judged by two researchers in parallel using no automation tools; a third researcher was used to settle disagreements between the two principal judges. No external protocol exists for this systematic review; the systematic review was conducted exactly as described above. PRISMA 2020 reporting guidelines for abstracts and manuscripts were followed, as shown in the Supporting Information (Section 7: S4 and S5 Figs).

### 2.1. Limitations of the systematic review

For each search term within each search year, only the first twenty results were browsed. Publications outside of the first twenty results were not identified using this method. Search engines return results in order of popularity/number of citations, so this review predominantly includes well-cited publications. Citation bias is therefore a possibility.

ScienceDirect, ResearchGate and Google Scholar were chosen for their global accessibility with the aim of reducing language bias and location bias. While they returned publications from every continent and in many languages, academic search engines may have reporting biases that are outside of the awareness and control of the authors.

The limitations of this systematic review are primarily a result of a small research team and limited budget. With increased resources, additional databases could have been searched at greater depths, and toll access reports could have been included.

### 2.2. Results of the systematic review

As shown in Fig 1, 189 publications were identified, of which 37 were not duplicates. Data was collected from 14 publications (Table 8). The remaining 23 reports were excluded from data collection (Table 9). From the included reports, 395 data points were collected.

**Table 8:** Publications subject to data collection.

Author(s)	Date	Title	Open access (OA)/Toll access (TA)	Accessed via	Date last accessed	Data points collected (Max: 53)
Brito *et al.* [29]	2023	Toxicity and antitumor activity of the water-soluble…	OA	Science Direct, Research Gate, Google Scholar	13/01/2025	27
Ezemagu *et al.* [17]	2023	Evaluating the Renal Toxicity…	OA	Research Gate, Google Scholar	13/01/2025	36
Attah *et al.* [18]	2022	*Moringa oleifera* seed at the interface…	OA	Research Gate, Google Scholar	13/01/2025	23
Dibua *et al.* [30]	2022	Assessment of Acute Toxicity…	OA	Research Gate, Google Scholar	13/01/2025	23
Yurre *et al.* [31]	2020	Evaluation of the Cardiac Effects of…	OA	Research Gate, Google Scholar	13/01/2025	29
Potestà *et al.* [32]	2019	Cytotoxic and apoptotic effects…	OA	Research Gate, Google Scholar	13/01/2025	14
Igbokwe *et al.* [33]	2018	Acute and Sub-Chronic Toxicities…	OA	Research Gate, Google Scholar	13/01/2025	41
Kim *et al.* [19]	2018	A 14-day repeated-dose oral toxicological…	OA	Science Direct, Research Gate, Google Scholar	13/01/2025	31
Idoko *et al.* [14]	2017	Sub-acute Toxicity Study of Defatted…	OA	Research Gate, Google Scholar	13/01/2025	40
Chinenyenwa & Godson [34]	2017	Bio-insecticidal efficacy of *Moringa*…	OA	Research Gate, Google Scholar	13/01/2025	15
Santana Silva *et al.* [35]	2017	Ecotoxicity of water-soluble lectin from *Moringa oleifera*…	OA	Science Direct, Research Gate, Google Scholar	13/01/2025	16
Ilyas *et al.* [15]	2016	Toxicological Assessment of…	OA	Research Gate, Google Scholar	13/01/2025	40
Ajayi *et al.* [36]	2016	Toxicological evaluation of…	OA	Research Gate, Google Scholar	13/01/2025	29
Ugochukwu *et al.* [16]	2016	Toxicological Studies on the Ethanol…	OA	Research Gate, Google Scholar	13/01/2025	31
				**Total data points collected:**		**395**

**Table 9:** Publications not subject to data collection.

Author(s)	Date	Title	Accessed via	Reason(s) excluded from data collection
Alves *et al.*	2024	Evaluation of Cytotoxicity and…	Research Gate, Google Scholar	Incompatible methodology: tested MO leaf; no testing of MO seed.
Alanazi *et al.*	2024	Effect of moringa seed extract in chlorpyrifos…	Research Gate, Google Scholar	Incompatible methodology: tested neuroprotective effects of MO seed.
Palomino-Pacheco *et al.*	2024	Assessment of oral toxicity of *Moringa oleifera* Lam aqueous…	Research Gate, Google Scholar	Incompatible methodology: tested MO leaf; no testing of MO seed.
Camilleri & Blundell	2024	A comprehensive review of the phytochemicals…	Science Direct, Research Gate, Google Scholar	Review.
Zhang *et al.*	2024	Evaluation of the feeding safety of…	Research Gate, Google Scholar	Incompatible methodology: tested MO leaf and stem; no testing of MO seed.
Bezerra *et al.*	2023	Antimalarial potential of Moringa…	Research Gate, Google Scholar	Review.
Pareek *et al.*	2023	*Moringa oleifera*: An Updated…	Research Gate, Google Scholar	Review.
Pop *et al.*	2022	A Comprehensive Review of Moringa…	Research Gate, Google Scholar	Review.
Melebary & Elnaggar	2022	Impact of *Moringa oleifera* leaf extract in reducing the effect of…	Research Gate, Google Scholar	Incompatible methodology: tested renoprotective and hepatoprotective effects of MO leaf.
Barros *et al.*	2022	Evaluation of acute toxicity, 28-day repeated dose…	Science Direct, Research Gate, Google Scholar	Toll access at time of identification and screening (January 2025).
Lopez-Martinez *et al.*	2021	Quality Control and Safety of Moringa	Research Gate, Google Scholar	Book chapter. Toll access at time of identification and screening (January 2025).
Popoola *et al.*	2020	A systematic review of pharmacological…	Research Gate, Google Scholar	Systematic review.
Olubunmi *et al.*	2020	Isolation, Characterisation and…	Research Gate, Google Scholar	Incompatible methodology: in silico/computational testing
Njan *et al.*	2018	Chronic toxicological evaluation and…	Research Gate, Google Scholar	Toll access at time of identification and screening (January 2025).
Moodley	2017	Acute toxicity of *Moringa oleifera* leaf…	Google Scholar	Incompatible methodology: tested MO leaf; no testing of MO seed.
El-Hak *et al.*	2017	Toxic effect of Moringa peregrina seeds on…	Research Gate, Google Scholar	Incompatible methodology: tested *Moringa peregrina* seeds.
Luz *et al.*	2017	cytotoxicity of the coagulant Moringa…	Science Direct, Google Scholar	Incompatible methodology: tested oncoprotective effects of MO lectin.
Okumu *et al.*	2016	Acute toxicity of the aqueous-methanolic…	Research Gate, Google Scholar	Incompatible methodology: tested MO leaf; no testing of MO seed.
Stohs & Hartman	2015	Review of the safety and Efficacy of…	Research Gate, Google Scholar	Review.
Osman *et al.*	2015	Assessment of acute toxicity and…	Research Gate, Google Scholar	Incompatible methodology: tested MO leaf; no testing of MO seed.
Olorundare *et al.*	2015	Acute and subacute toxicities of defatted…	Google Scholar	Toll access at time of identification and screening (January 2025).
Al-Anizi *et al.*	2014	Toxicity assessment and modelling of *Moringa oleifera*…	Science Direct, Research Gate, Google Scholar	Toll access at time of identification and screening (January 2025).
Priyadarshani	2014	Effect of *Moringa oleifera*…	Google Scholar	Incompatible methodology: tested MO leaf; no testing of MO seed.

**Fig 1:**
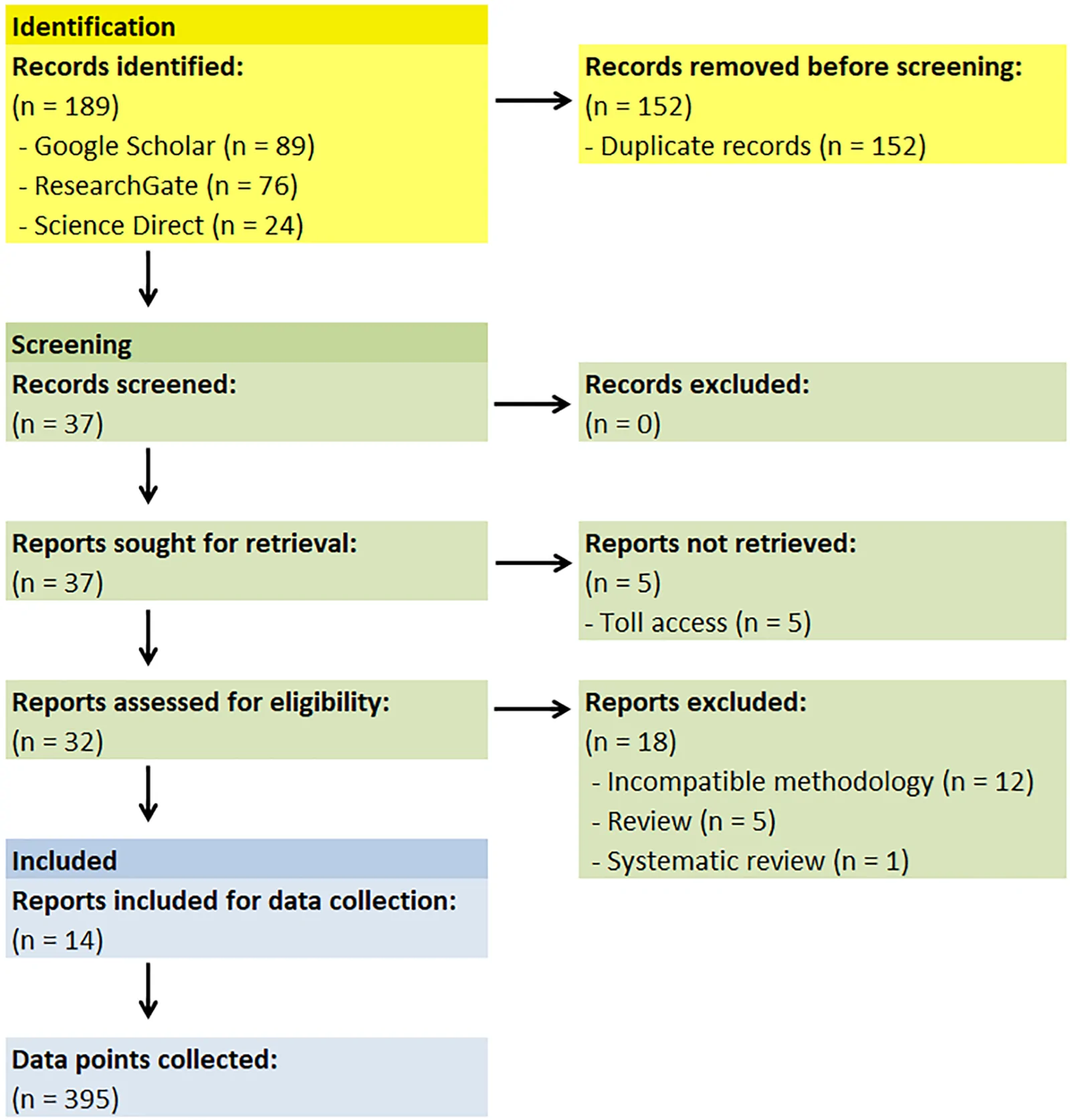
PRISMA (2020) flow diagram displaying the results of the systematic review.

As shown in Table 10, Kim *et al.* [19] were the only included report to declare financial conflicts of interest: one author is an inventor of patented MO seed extract technologies and has financial interests in a private enterprise affiliated with a second author. No declared or apparent undeclared conflicts of interest were found in any other report, which were all conducted by university researchers.

**Table 10:** Results of conflict of interest assessments.

Publication	Conflict of interest judgement	Reasoning for conflict of interest judgement
Brito *et al.* [29]	No notable concern about conflict of interest	No declared conflict of interest; no apparent undeclared conflict of interest
Ezemagu *et al.* [17]	No notable concern about conflict of interest	No declared conflict of interest; no apparent undeclared conflict of interest
Attah *et al.* [18]	No notable concern about conflict of interest	No declared conflict of interest; no apparent undeclared conflict of interest
Dibua *et al.* [30]	No notable concern about conflict of interest	No declared conflict of interest; no apparent undeclared conflict of interest
Yurre *et al.* [31]	No notable concern about conflict of interest	No declared conflict of interest; no apparent undeclared conflict of interest
Potestà *et al.* [32]	No notable concern about conflict of interest	No declared conflict of interest; no apparent undeclared conflict of interest
Igbokwe *et al.* [33]	No notable concern about conflict of interest	No declared conflict of interest; no apparent undeclared conflict of interest
Kim *et al.* [19]	Notable concern about conflict of interest	Financial conflict of interest declared
Idoko *et al.* [14]	No notable concern about conflict of interest	No declared conflict of interest; no apparent undeclared conflict of interest
Chinenyenwa & Godson [34]	No notable concern about conflict of interest	No declared conflict of interest; no apparent undeclared conflict of interest
Santana Silva *et al.* [35]	No notable concern about conflict of interest	No declared conflict of interest; no apparent undeclared conflict of interest
Ilyas *et al.* [15]	No notable concern about conflict of interest	No declared conflict of interest; no apparent undeclared conflict of interest
Ajayi *et al.* [36]	No notable concern about conflict of interest	No declared conflict of interest; no apparent undeclared conflict of interest
Ugochukwu *et al.* [16]	No notable concern about conflict of interest	No declared conflict of interest; no apparent undeclared conflict of interest

Research quality assessments returned a variety of judgements: 4 of 14 reports received a “highest quality” judgement; one report received a “higher quality” judgement; three reports received “lower quality” judgements, and; six reports received “lowest quality” judgements. Three of four “highest quality” reports were non-rodent studies [29,34,35]; standards and ethical guidelines for research on human cell lines and the embryos of insects and fish were less rigorous than those of rodent studies. The remaining 11 reports were rodent studies, of which only two reports were of “highest quality” or “higher quality”. Inappropriate experimental design (Q-Q2) and insufficient group sizes (Q-Q3) were common causes of quality loss in rodent studies. Conversely, analysis was largely transparent (Q-Q7), appropriate (Q-Q8) and applied evenly across groups (Q-Q9). Signalling question judgements and overall quality judgements are shown in Table 11.

**Table 11:** Results of research quality assessments.

	Signalling questions for research quality assessment											
Publication	Q-Q1	Q-Q2	Q-Q3	Q-Q4	Q-Q5	Q-Q6	Q-Q7	Q-Q8	Q-Q9	Q-Q10	QS	QJ
Brito *et al.* [29]^a^	U	Y	Y	Y	Y	Y	Y	Y	Y	Y	19	**Highest quality**
Ezemagu *et al.* [17]^a^	N	N	N	N	Y	U	Y	Y	Y	Y	11	**Lowest quality**
Attah *et al.* [18]^a^	U	N	N	Y	Y	Y	Y	Y	Y	Y	15	**Lower quality**
Dibua *et al.* [30]^a^	U	N	Y	Y	Y	Y	N	U	U	N	11	**Lowest quality**
Yurre *et al.* [31]^a^	U	N	U	Y	Y	U	Y	Y	Y	Y	15	**Lower quality**
Potestà *et al.* [32]^b^	Y	U	Y	Y	Y	Y	Y	Y	Y	Y	19	**Highest quality**
Igbokwe *et al.* [33]^a^	U	N	N	Y	Y	U	Y	Y	Y	U	13	**Lowest quality**
Kim *et al.* [19]^a^	Y	N	Y	Y	Y	Y	Y	Y	Y	U	17	**Higher quality**
Idoko *et al.* [14]^a^	U	N	N	N	Y	U	Y	Y	Y	Y	12	**Lowest quality**
Chinenyenwa & Godson [34]^b^	Y	Y	Y	Y	Y	Y	Y	Y	Y	Y	20	**Highest quality**
Santana Silva *et al.* [35]^b^	Y	Y	Y	Y	Y	Y	Y	Y	Y	Y	20	**Highest quality**
Ilyas *et al.* [15]^a^	Y	N	N	Y	Y	Y	Y	Y	Y	U	15	**Lower quality**
Ajayi *et al.* [36]^a^	U	N	N	Y	Y	U	Y	Y	Y	Y	14	**Lowest quality**
Ugochukwu *et al.* [16]^a^	U	N	N	N	Y	Y	Y	Y	Y	U	12	**Lowest quality**

^a^Rodent study.

^b^Non-rodent study.

Risk of bias assessments also returned a variety of judgements: 6 of 14 reports received “low risk of bias” judgements, two reports received “some concerns” judgements and six reports received “high risk of bias” judgements. Bias risk was most commonly detected in bias domain 3, due to the omission of relevant data when drawing conclusions (B-Q3.3). Consequently, a number of concluding statements (from discussion, conclusion and abstract sections) were not supported by reported data. Dibua *et al.* [30] stated MO seed oil was “*relatively safe both for nutritional and medicinal uses*”, despite reporting “*dose-dependent necrosis*” of liver tissue in all test groups. Kim *et al.* [19] reported a no-observable-adverse-effect level (NOAEL) of 257 mg/kg bw/d for MO seed extract, despite reporting respiratory pattern symptoms (i.e., “*irregular respiration*” and “*moist rales*”) in rats dosed at 257 mg/kg bw/d. Ilyas *et al.* [15] stated “*there was no major difference in body weight of the control and treated animals*”, despite reporting dose-dependent decreases in weight gain in all test groups. Ajayi *et al.* [36] stated the “*seed extracts of M. oleifera could boost immunity and offer hepatoprotective effects*”, despite reporting significantly decreased aspartate aminotransferase (AST) and alkaline phosphatase (ALP) in all test groups and “*moderate hepatic necrosis*” at 200 mg/kg bw/d of MO seed extract. Finally, Ugochukwu *et al.* [16] stated MO seed “*did not perturb the levels of key biological markers used in assessing liver and haematological functions*”, despite reporting a decrease in AST from 170.20 U/L to 69.00 U/L and a significant increase in platelet count from 165.00 (10^9^/L) to 504.75 (10^9^/L). Selective reporting of data may indicate a deliberate or subconscious willingness to overestimate the safety and utility of MO seed consumption. In the case of Kim *et al.* [19], overestimation of MO seed safety limits may align with the researchers’ financial conflicts of interest. In other reports, researchers may be influenced by trends in pharmacognosy, folkloric trust in *M. oleifera* products or unapparent conflicts of interest. Bias domain judgements and overall risk of bias judgements are shown in Table 12.

**Table 12:** Results of risk of bias assessments.

	Judgements for each bias domain			Overall risk of bias judgement
Publication	B-J1	B-J2	B-J3	B-J4
Brito *et al.* [29]	Low risk of bias	Low risk of bias	Low risk of bias	**Low risk of bias**
Ezemagu *et al.* [17]	High risk of bias	Low risk of bias	Low risk of bias	**High risk of bias**
Attah *et al.* [18]	Low risk of bias	Low risk of bias	Low risk of bias	**Low risk of bias**
Dibua *et al.* [30]	Some concerns	Some concerns	Some concerns	**High risk of bias**
Yurre *et al.* [31]	Low risk of bias	Low risk of bias	Low risk of bias	**Low risk of bias**
Potestà *et al.* [32]	Low risk of bias	Low risk of bias	Low risk of bias	**Low risk of bias**
Igbokwe *et al.* [33]	Some concerns	Some concerns	Low risk of bias	**Some concerns**
Kim *et al.* [19]	Low risk of bias	Some concerns	High risk of bias	**High risk of bias**
Idoko *et al.* [14]	Some concerns	Low risk of bias	Some concerns	**Some concerns**
Chinenyenwa & Godson [34]	Low risk of bias	Low risk of bias	Low risk of bias	**Low risk of bias**
Santana Silva *et al.* [35]	Low risk of bias	Low risk of bias	Low risk of bias	**Low risk of bias**
Ilyas *et al.* [15]	Low risk of bias	Some concerns	High risk of bias	**High risk of bias**
Ajayi *et al.* [36]	Some concerns	Low risk of bias	High risk of bias	**High risk of bias**
Ugochukwu *et al.* [16]	Some concerns	Some concerns	Some concerns	**High risk of bias**

Judgements for conflict of interest, overall research quality and overall risk of bias are collected in Table 13 to give a comprehensive impression of each included report. Four reports received “highest quality” and “low risk of bias” judgements; these were the most reliable of the included reports. Brito *et al.* [29] were the only rodent study to receive both of these judgements. Four reports received “lowest quality” and “high risk of bias” judgements; these were the least reliable of the included reports. In compliance with standard systematic review practices, reports with high reliability were given increased weight in the discussion and reports with low reliability were given reduced weight in the discussion. Research quality and risk of bias are incorporated into the data synthesis of the systematic review using a strategy of inclusion and narrative discussion, as opposed to a strategy of report exclusion or synthesis limitation.

**Table 13:** Combined results of assessments for conflict of interest, overall research quality and overall risk of bias.

Publication	Conflict of interest judgement	Overall research quality judgement (QJ)	Overall risk of bias judgement (B-J4)
Brito *et al.* [29]^a^	No notable concern about conflict of interest	Highest quality	Low risk of bias
Ezemagu *et al.* [17]^a^	No notable concern about conflict of interest	Lowest quality	High risk of bias
Attah *et al.* [18]^a^	No notable concern about conflict of interest	Lower quality	Low risk of bias
Dibua *et al.* [30]^a^	No notable concern about conflict of interest	Lowest quality	High risk of bias
Yurre *et al.* [31]^a^	No notable concern about conflict of interest	Lower quality	Low risk of bias
Potestà *et al.* [32]^b^	No notable concern about conflict of interest	Highest quality	Low risk of bias
Igbokwe *et al.* [33]^a^	No notable concern about conflict of interest	Lowest quality	Some concerns
Kim *et al.* [19]^a^	Notable concern about conflict of interest	Higher quality	High risk of bias
Idoko *et al.* [14]^a^	No notable concern about conflict of interest	Lowest quality	Some concerns
Chinenyenwa & Godson [34]^b^	No notable concern about conflict of interest	Highest quality	Low risk of bias
Santana Silva *et al.* [35]^b^	No notable concern about conflict of interest	Highest quality	Low risk of bias
Ilyas *et al.* [15]^a^	No notable concern about conflict of interest	Lower quality	High risk of bias
Ajayi *et al.* [36]^a^	No notable concern about conflict of interest	Lowest quality	High risk of bias
Ugochukwu *et al.* [16]^a^	No notable concern about conflict of interest	Lowest quality	High risk of bias

^a^Rodent study.

^b^Non-rodent study.

The complete dataset, including all collected data points and all data pertaining to assessments for conflict of interest, research quality and risk of bias, is available via open-access data repository (https://doi.org/10.5061/dryad.6q573n6f3) [37].

## 3. Discussion

### 3.1. Background information

An understanding of MO seed phytochemistry is beneficial when interpreting the results of toxicology reports. In context of water treatment, proteins are the most studied primary metabolites, due to the involvement of *Moringa oleifera* cationic proteins (MOCPs) in coagulation, flocculation and sedimentation (CFS). Lipids are also well-studied primary metabolites, due to their use as ben oil and biofuel. Secondary metabolites, such as organosulfur, phenolic and alkaloid compounds, have only been studied in a pharmaceutical context to date. A number of these phytochemicals are discussed below, with emphasis on parameters pertaining to MO seed toxicology and coagulant preparation. The aim of this section is to update readers on recently-discovered phytochemicals derived from MO seeds, and to encourage researchers to study these phytochemicals in a toxicological context, where necessary. The systematic review of included reports begins in Section 3.2.

#### 3.1.1. Mustard oil bomb reaction.

The mustard oil bomb (MOB) reaction is the characteristic antifeedant system of Brassicale species, such as MO. In this reaction, glucosinolates are hydrolysed by the enzyme myrosinase, producing isothiocyanates. In intact Brassicale plant tissues, glucosinolates and myrosinase are compartmentalised. When predated by a herbivore, chewing mixes the substrate and enzyme, saliva facilitates hydrolysis, and isothiocyanates are produced in the mouth [38]. The pungent taste of certain cruciferous vegetables can be attributed to this reaction; wasabi is perhaps the best example. In laboratory settings, this process is simulated when MO seeds are processed and subject to water-based solvents.

Myrosinase is a mandatory component of the MOB reaction, but it is also susceptible to heat inactivation; a susceptibility that is exploited in cooking. Ghawi *et al.* [39] reviewed the thermal stability of myrosinase from eight Brassicale species: in seven of eight cases, myrosinase was inactivated at 30–65°C (303.15–338.15 K). The outlier—myrosinase from intact and flaked rapeseed seeds—was inactivated at 90°C (363.15 K). The precise thermostability of MO myrosinase is unknown, but boiling is known to preserve glucosinolate content and prevent the production of isothiocyanates [40]. Therefore, elevated temperatures (anywhere from 30–100°C / 303.15–373.15 K) are theorised to inactivate MO myrosinase and “defuse” the MOB reaction. In context of toxicology, methods of MO seed processing that include elevated temperatures can be referred to “hot preparations”. Conversely, processing methods that do not exceed 30°C (303.15 K) can be referred to as “cold preparations”.

From here, cold preparations can be further categorised into “cold–wet preparations” and “cold–dry preparations”. Cold–wet preparations are conducted at < 30°C (303.15 K) and involve the *in vitro* addition of water to the MO seed powder or extract (e.g., aqueous or saline extractions). In this scenario, isothiocyanates are produced *in vitro*, where they may degrade if not dried or frozen [41]. In toxicity studies, a whole or diminished isothiocyanate dose is then administered to the test organisms, depending on the degree of preservation. Cold–dry preparations are conducted at < 30°C (303.15 K) and do not involve the *in vitro* addition of water (e.g., MO seed powders or extracts made with organic solvents). In this scenario, glucosinolate content is preserved until dosing, whereby water in the test subject’s body facilitates *in vivo* production of isothiocyanates.

Cold–dry preparations most closely resemble the MOB reaction in nature, although there is a notable difference in rodent studies. MO seed products are commonly administered via gavage, whereby stomach acid is the medium for the MOB reaction. Glucosinolates are more likely to be hydrolysed into nitrile products at low pH (< 4), as shown in Fig 2, and the pH of gastric acid in rats and mice is 3.0–4.0 [42,43]. Naturally, saliva facilitates the production of isothiocyanates in the mouth, whereas gavage feeding promotes the production of isothiocyanates and nitriles in the stomach.

**Fig 2:**
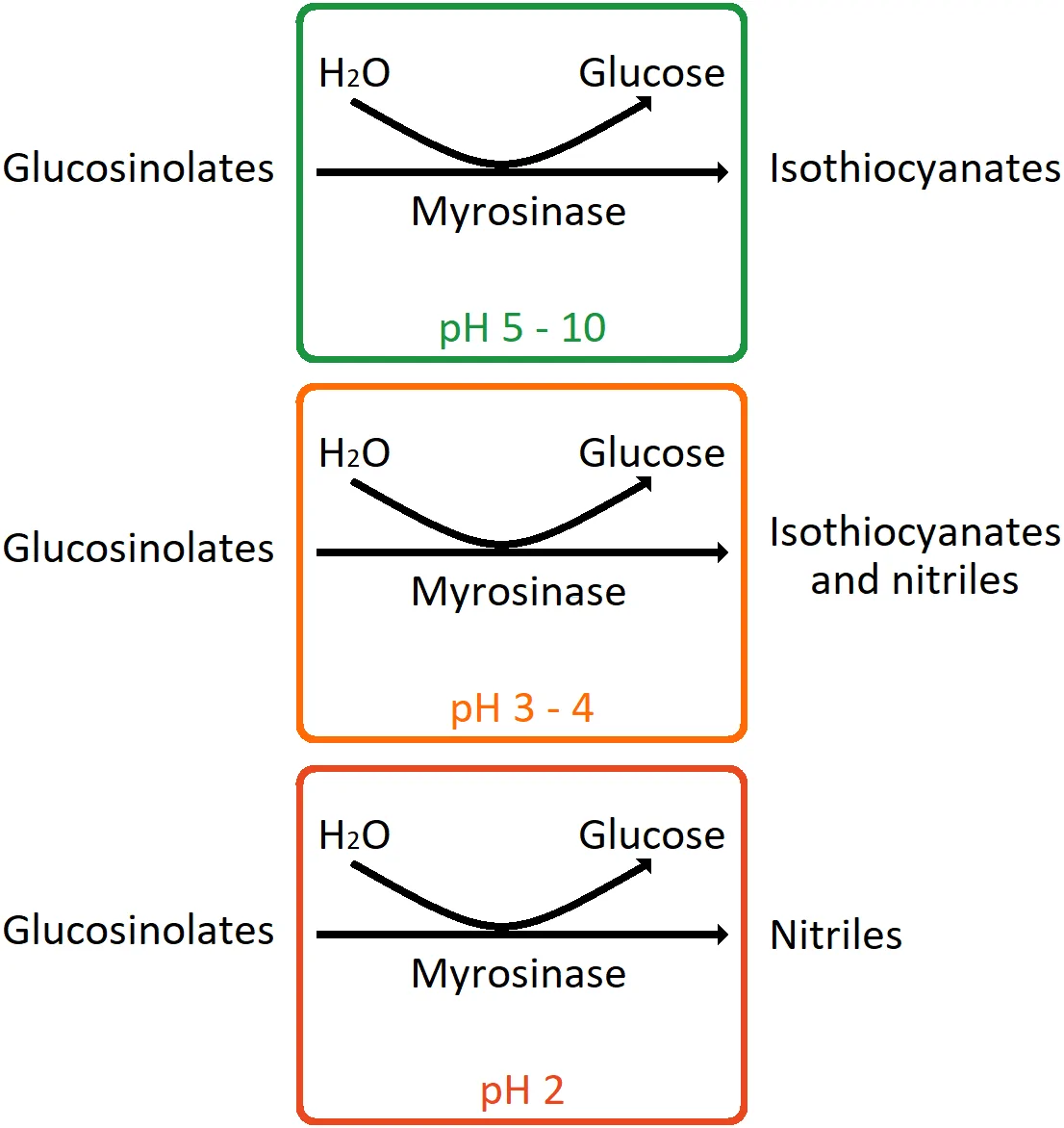
The mustard oil bomb reaction. Notice the pH-dependent production of isothiocyanates and/or nitriles from glucosinolates.

Nitriles are organic compounds containing the cyano functional group (−C ≡ N); inorganic compounds containing this group are known as cyanides. Nitriles have been isolated from MO products in the form of cyanogenic glycosides, namely niaziridin, niazirin and niazidin [44,45]. The EU Panel on Contaminants in the Food Chain (CONTAM) recommends a NOAEL of ≤ 20 μg CN/kg bw for acute doses. Regarding chronic exposure to cyanogenic glycosides, they state the “*available evidence from animal and human studies does not allow the derivation of a chronic health-based guidance value*” [46]. Nitrile production via gavage feeding of glucosinolates is a theoretical route of toxicity that requires additional research.

#### 3.1.2. Organosulfur compounds – Glucosinolates.

Glucosinolates are sulphur-containing glycosides that act as substrate for the MOB reaction. Glucomoringin (4-(α-L-rhamnopyranosyloxy)benzyl glucosinolate; 4RBGS) is the predominant glucosinolate in seeds of domesticated MO cultivars and glucosoonjnain (4-(glucopyranosyloxy)benzyl glucosinolate; 4GBGS) is predominant in wild cultivars [47,48].

Glucosinolates are water-soluble and can be reduced in concentration in cruciferous vegetables via boiling. It is unclear whether they are unstable at boiling temperatures, defuse into the boiling water or are hydrolysed by myrosinase on contact with water [41]. Glucosinolates are occasionally referred to as “goitrogenic glycoside”, because their products can disrupt thyroid hormone production in animal models [49].

#### 3.1.3. Organosulfur compounds – Isothiocyanates.

Moringin (4-(α-l-rhamnopyranosyloxy)-benzyl isothiocyanate; 4RBITC) and soonjnain are the respective isothiocyanate products of glucomoringin and glucosoonjnain hydrolysis [47]. Moringin is weakly water-soluble and unstable in water, decaying into several water-soluble products, such as rhamnobenzyl alcohol, di-rhamnobenzyl thiourea and 4-[(α-l-rhamnosyloxy)benzyl]thiocyanate. These products display weak bioactivity when compared to moringin [41]. Soonjnain was discovered in 2018 and its properties are not yet fully explored [48]. Isothiocyanate testing is largely concerned with anti-inflammatory, anti-oxidative and anticancer properties, so limited toxicology data is available for moringin or soonjnain.

#### 3.1.4. Organosulfur compounds – Thiocarbamates.

Thiocarbamates are organosulfur compounds with insecticidal, herbicidal and fungicidal properties. Many carbamates and thiocarbamates are potent acetylcholinesterase inhibitors. Niazimicin(e), niaziminin(e) and niazinin(e) are thiocarbamate glycosides isolated from MO; their capacity for acetylcholinesterase inhibition has not yet been tested and their physiochemical properties are unknown [44]. Costa *et al.* [50] suggest “*the addition of methanol or ethanol to isothiocyanate is considered a pathway to the synthesis of thiocarbamates glycosides in Moringa*”. A number of publications report thiocarbamates in organic solvent extracts of MO seeds, so this theory requires further investigation [51–53].

#### 3.1.5. Lipids – Fatty acids.

MO seeds are rich in long-chain and very-long-chain fatty acids with an oil content of 33–42% by weight [54,55]. In a review of 14 publications by Al-Jadabi *et al.* [56], unsaturated fatty acids were found to dominate the lipid fraction at mean content of 73.1% oleic acid (C18:1, ω − 9), 2.2% gadoleic acid (C20:1, ω − 11), 1.9% palmitoleic acid (C16:1, ω − 7), 1.1% nurvonic acid (C24:1, ω − 9) and 0.9% linoleic acid (C18:2, ω − 6). In the same review, mean saturated fatty acid contents were 6.6% palmitic acid (C16:0), 5.7% behenic acid (C22:0), 4.8% stearic acid (C18:0), 3.0% arachidic acid (C20:0), 1.0% lignoceric acid (C24:0) and 0.8% cerotic acid (C26:0). Nine additional fatty acids were identified at < 0.4%.

Fatty acids are insoluble in water and soluble in non-polar solvents, such as n-hexane; they are less dense than water and form a floating scum. Thermal decomposition of oleic acid and stearic acid occurs at 175–300 °C (448.15–573.15 K) and 225–325°C (498.15–598.15 K), respectively [57,58]. Thermostability data for other fatty acids is not readily available, though similar decomposition ranges are expected. In excess, saturated fatty acids contribute to metabolic syndrome conditions. Acute oversaturation of the body’s adipose tissue with saturated or unsaturated fatty acids can also cause lipotoxicity [59]. Lipotoxicity has degenerative effects on non-adipose tissues of the heart, liver, kidneys, pancreas and skeletal muscle when they attempt to store surplus fatty acids [60].

#### 3.1.6. Lipids – Fatty acid methyl esters.

Fatty acid methyl esters (FAMEs) have high energy density and are utilised in biofuel production. FAMEs occur naturally in MO seeds and can be synthesised in higher concentrations via transesterification of fatty acids and methanol [61]. Olubunmi *et al.* [62] reported the isolation of methyl trans-vaccenate (6.5%), methyl palmitate (2.5%) and methyl stearate (1.3%) in an ethanolic extract of MO seeds. In an n-hexane extract of MO seed oil, Bassey *et al.* [63] reported methyl oleate (7.9%), methyl palmitate (1.2%) and methyl cis-11-eicosenoate (1.1%).

Like fatty acids, FAMEs are insoluble in water, soluble in non-polar solvents and less dense than water. FAMEs are marginally more thermostable than fatty acids, exhibiting thermal decomposition at > 325 °C (598.15 K) [64,65]. Limited toxicity data is available for FAMEs.

#### 3.1.7. Lipids – Triglycerides.

Triglycerides are esters of glycerol and fatty acids. Fatty acids are a readily available source of energy, whereas triglycerides act as an energy store. Polyunsaturated triglycerides are common in MO seed oil due to the prevalence of oleic acid (O). Other common constituents of MO triglycerides include linoleic (L), gadoleic (G), palmitic (P) and stearic (S) acid. In a study of MO seed extracts, Abdulkarim *et al.* [66] reported the following triglyceride profile: Triolein (OOO) (36.7%), POO + SOL (12.4%), SOO (11.4%), OOA (7.7%), POL (5.0%), OOG (4.1%) and seven additional fractions at ≤ 2%. Triolein (OOO) was also reported by Olubunmi *et al.* [62], alongside trivaccenin (PPP).

Triglycerides share properties with their fatty acid constituents: they are insoluble in water, soluble in non-polar solvents and less dense than water. Thermal decomposition of triglycerides into glycerol and free fatty acids occurs at 250–300 °C (523.15–573.15 K) [67,68]. Elevated serum triglycerides (hypertriglyceridemia) is a risk factor of cardiovascular disease and pancreatitis [69].

#### 3.1.8. Lipids – Phytosterols.

Phytosterols include plant sterols and stanols. They are amphipathic lipids that are structurally similar to cholesterol. In plants, they function like cholesterol; when ingested by animals, they complete for absorption with cholesterol in the intestines, lowering serum cholesterol concentration and reducing the risk of cardiovascular disease. Avenasterol, β-sitosterol, campesterol, stigmasterol and stigmastanol are major phytosterol components of MO seed oils [70,71].

As amphipathic lipids, phytosterols are soluble in both polar and non-polar solvents. They are also known to form micelles, which can be colloidal [72]. Phytosterols are stable at 100°C (373.15 K), and thermal decomposition has been demonstrated at 180°C (453.15 K) and 200°C (473.15 K) [73,74].

#### 3.1.9. Phenolic compounds – Flavonoids and flavonols glycosides.

MO products are known to include a number of flavonols and flavanols. Key flavonols include quercetin, kaempferol and myricetin, and key flavanols include catechin and epicatechin [75]. Flavanoids are soluble in polar solvents, reduced in concentration by cooking temperatures and are generally thought to be safe in moderation [76,77].

A large variety of quercetin and kaempferol glycosides have also been isolated from MO products [78,79]. Flavonol glycosides are thought to be more bioavailable than basic flavonols. Hydrolysis of flavonols glycosides is catalysed by ubiquitous glycosidases, producing flavonols and sugars [80].

#### 3.1.10. Phenolic compounds – Phenolic acids.

A large variety of phenolic acids have been reported in MO products, including caffeic, ferulic, gallic, isoferulic, protocatechuic, sinapinic, syringic and vanillic acid [71]. Gharsallah *et al.* [81] identified ferulic and caffeic acid as the predominant phenolic acids at 22.9 and 4.3 mg/kg of MO seed oil, respectively. Ferulic and caffeic acid are soluble in polar solvents and both are denser than water.

#### 3.1.11. Phenolic compounds – Lignans.

Lignans are polyphenolic compounds with antifeedant properties. Research on lignans from MO seeds is limited, but a number of lignans and lignan glycosides have been isolated from MO leaves, including secoisolariciresinol, 7-hydroxysecoisolariciresinol, 7-oxomatairesinol, epipinoresinol, medioresinol and isolariciresinol [82,83].

Lignans are soluble in polar solvents, denser than water, thermotolerant to 100 °C (373.15 K) when dry, but undergo thermal decomposition at < 100 °C (373.15 K) with high moisture content [84]. Phytoestrogens are plant-derived chemicals with structural similarities to 17-β-estradiol. Lignans are often listed alongside the well-studied phytoestrogens (isofalvones, prenylflavonoids and coumestans), but there is little evidence to show estrogenic activity in lignans [85]. Lignan bioavailability is also believed to be influenced by the composition of gut microbiota [86].

#### 3.1.12. Phenolic compounds – Tannins.

Like lignans, tannins are polyphenolic compounds with antifeedant properties. Tannins bind protein by interacting with specific amino acids. In doing so, they modify protein folding to inhibit enzyme functionality and change protein solubility [87]. Manan & Mohammed [88] reported tannin concentrations of 0.89 mg/g in MO seeds, representing ~8.7% of total phenolic content.

Unbound tannins are water-soluble, whereas the solubility of tannin–protein complexes depends on the properties of the modified protein [89]. Tannins are denser than water and undergo thermal decomposition at ~300 °C (573.15 K) [90]. Precipitation of dietary protein and inhibition of digestive enzymes are known antinutritional actions of tannins. Antinutrition from a high-tannin diet can result in slower weight gain and increases food consumption in animal models [91]. Ojo [92] states that “i*n humans, no evidence of toxicity arising from the consumption of excessive amounts of legume tannins has ever been reported*”.

#### 3.1.13. Alkaloids.

Plant alkaloids are a diverse class of nitrogen-containing compounds studied for both medicinal and toxicological purposes. N, α-L-rhamnopyranosyl vincosamide is the most frequently isolated alkaloid in MO products, though alkaloid glycosides are also reported: namely, marumoside A and marumoside B [93,94]. Two isothiocyanate-derived thioketone alkaloids—named spiroleiferthione A and oleiferthione A—were recently discovered in MO seeds [95]. Alkaloids are often considered to exist in low concentrations in MO products, but this assumption was challenged by Omokpariola *et al.* [96] who reported an alkaloid yield of 5.85% in an ethanol extract of MO seed.

Alkaloids have low solubility in water and are soluble in polar organic solvents, whereas alkaloid salts are water-soluble [97]. Thermal stability and density of MO-derived alkaloids have not been studied and generalisations cannot be made due to the diversity of alkaloid compounds. Likewise, the bioactive effects of MO-derived alkaloids have only been studied in a pharmaceutical context; therefore, toxicological data is unavailable.

### 3.2. Purpose of toxicological research

Among the included reports, there was general agreement that exposure to MO products—through food, pharmaceuticals and water treatment—is rapidly accelerating. The prevalence of MO research in tropical and sub-tropical regions suggests this is true. It was the view of many researchers that the evidence base for MO safety is too narrow, inconsistent and/or unstandardised in light of the tree’s rapid adoption. These concerns were the impetus for an influx of toxicological studies over the past decade (~85% of online literature regarding MO toxicology was published from 2014–2024).

In 2016, Ilyas *et al.* [15] stated “*medicinal plants are blindly used considering only their beneficial aspects, while toxicity or toxic doses are ignored.*”, and Ugochukwu *et al.* [16] stated “*herbal products fortified with M. oleifera parts are often not screened to ascertain their toxicological properties and safe-dose levels*”. In response to the consumption of raw MO seeds in 2017, Idoko *et al.* [14] stated “*caution must be exercised to avert the possible toxic effect of the plant*”. In 2018, Kim *et al.* [19] described safety studies as “*limited*” and highlighted a “*lack of biochemical characterization and standardization*”. In 2022, Attah *et al.* [18] stated MO seed usage “*has continued to gain momentum across different regions of the world without any attention to its potential toxicities*”. In 2023, Ezemagu *et al.* [17] described “*an urgent need to standardize*” due to concerns of renal failure from MO seed consumption. Similar warnings are made year-on-year, reiterating a need for standardised testing and definitive safety levels.

### 3.3. Acquisition and storage of *M. oleifera* seeds

MO seeds were acquired locally in 12 of 14 reports, demonstrating the wide availability of the tree [14–18,29–31,33–36]. Researchers in Italy and the USA obtained seeds from Cameroon and Jamaica, respectively [19,32]. Species verification was common, with 11 of 14 research groups consulting a taxonomist or herbarium [14–18,29–31,33,35,36].

Ezemagu *et al.* [17] were the only researchers to declare the age of their MO seeds: between harvest and testing, seeds were dried in the shade for two weeks. Igbokwe *et al.* [33] stated their MO seeds were harvested in June 2013, but it was unclear when testing occurred so the age of their seeds could not be determined. The phytochemical composition of plant materials can change as they age, so MO seed age was considered a confounding factor in risk of bias assessments. This factor can affect an independent variable (toxic phytochemical dose) and dependant variables (severity of toxicities) simultaneously. Failure to declare MO seed age was a common bias concern.

In two reports, MO seeds were reduced to a powder upon receipt, whereby the powders were stored in an air-tight containers pending further processing [15,34]. Yurre *et al.* [31] were the only researchers to freeze their seeds upon receipt. Seed storage conditions were not disclosed in other reports.

### 3.4. Processing of *M. oleifera* seeds

MO seed processing method was a complexed independent variable with limited standardisation. Complexity arose from the degree of processing (primary, secondary and tertiary), the choice of solvent (e.g., water, ethanol, methanol) and from processing temperature, which can alter the phytochemical profile of MO seeds, as discussed in Section 3.1.1.

Care was taken to thoroughly examine the methodologies of all included reports, as displayed in Table 14. Ezemagu *et al.* and Idoko *et al.* [14,17] were the only researcher groups to test the toxicity of primary-processed MO seed powders, although the former also conducted aqueous and ethanolic extractions to perform qualitative analysis of MO seed phytochemicals. Secondary-processed extracts were tested in 9 of 14 reports and solvent choice was inconsistent: solvents were aqueous (n = 3), ethanol (n = 2), aqueous-ethanol (n = 1), methanol (n = 1), aqueous-methanol (n = 1) and n-hexane (n = 1) [15,16,18,19,30,32–34,36]. Three researcher groups produced a tertiary-processed MO seed lectin [29,31,35]. By following the methodology of Coelho *et al.* [98], a standardised lectin was produced in each instance. Theoretical dose types were also included in the right-most column of Table 14. Classifications were based on the exposure of MO seeds to water and elevated temperature (> 30°C / 303.15 K), and by considering which order these events occurred (if at all). Cold–wet preparations (i.e., active myrosinase/*in vitro* isothiocyanate production) were tested in 8 of 14 reports [16–19,32–34,36]; hot preparations (i.e., inactivated myrosinase/no isothiocyanate production) were tested in 3 of 14 reports [14,15,32]. Purified MO seed lectin was excluded from classification because it does not include myrosinase, glucosinolate or isothiocyanate. The processing methods carried out by Ajayi *et al.*, Ezemagu *et al.*, Igbokwe *et al.* and Ugochukwu *et al.* [16,17,33,36] were considered cold–dry (i.e., active myrosinase/ *in vivo* isothiocyanate production) until dose administration, whereby an aqueous vehicle was used and MO seed was wetted *in vitro*. In these instances, there is the possibility of *in vitro* and *in vivo* isothiocyanate production, as the MOB reaction may not occur instantaneously.

**Table 14:** Methods of processing *M. oleifera* seed and theoretical dose type.

Report	Primary processing	Defatting	Secondary processing	Tertiary processing	Maximum temperature	Aqueous vehicle^a^	Theoretical dose type
Brito *et al.* [29]	Crushing	No	Aqueous extraction; ammonium sulfate fractionation	Chitin-binding column	28°C (301.15 K)	No (PBS, IP injection)	N/A (purified MO seed lectin)
Ezemagu *et al.* [17]	Blending	No	N/A (extracts used for chemistry)	N/A	Room temperature^**b**^	Yes	cold–wet^c^ seed powder
Attah *et al.* [18]	Powdered	Tested whole & defatted	Aqueous–methanol extraction (1:1)	N/A	Room temperature^**b**^	Yes	cold–wet aqueous–methanol extract
Dibua *et al.* [30]	Crushing & grinding	No	n-hexane extraction	N/A	Room temperature	No (liquid oil)	N/A (purified MO seed lipids)
Yurre *et al.* [31]	Powdered	No	Aqueous extraction; ammonium sulfate fractionation	Chitin-binding column	28°C (301.15 K)	Yes	N/A (purified MO seed lectin)
Potestà *et al.* [32]	Grinding	No	Aqueous extraction	N/A	Boiled and frozen extracts	N/A	hot and cold–wet aqueous extracts
Igbokwe *et al.* [33]	Powdered	No	Ethanol extraction	N/A	Room temperature^**b**^	Yes	cold–wet^C^ ethanol extract
Kim *et al.* [19]	Grinding	No	Aqueous–ethanol extraction	N/A	37°C (310.15 K) following aqueous extraction	Yes	cold–wet aqueous–ethanol extract
Idoko *et al.* [14]	Pulverised	Yes	N/A	N/A	“*warm water*”; 40°C (313.15 K) baking	N/A	hot seed powder
Chinenyenwa & Godson [34]	Powdered	No	Aqueous extraction	N/A	25°C (298.15 K)	N/A	cold–wet aqueous extract
Santana Silva *et al.* [35]	Powdered	No	Aqueous extraction; ammonium sulfate precipitation	Chitin-binding column	25°C (298.15 K)	N/A	N/A (purified MO seed lectin)
Ilyas *et al.* [15]	Blending	No	Aqueous extraction	N/A	“*warm water*” used in extraction	Yes	hot aqueous extract
Ajayi *et al.* [36]	Blending	No	Methanol extraction	N/A	“*cold extraction*”	Unclear	cold–dry or cold–wet^C^ methanol extract
Ugochukwu *et al.* [16]	Crushing & blending	No	Ethanol extraction	N/A	Room temperature	Yes	cold–wet^C^ ethanol extract

^a^Vehicle refers to the media in which the dose is administered to test organisms.

^b^Room temperature assumed in lieu of temperature data.

^c^Dose prepared in “cold–dry” manner and made “cold–wet” when mixed with aqueous vehicle.

Imprecise language was used by a number of researchers, most commonly when reporting MO seed processing methods. The term “warm” was most consequential to this review, so effort was made to define it. Ntoumani *et al.* [99] reviewed studies of thermoregulatory responses to whole-body liquid immersion in humans. Among other definitions, they concluded that a temperature range of 38–43°C (311.15–316.15 K) is commonly considered “warm” for liquids. This definition was used in place of imprecise language when necessary, but precise reporting is encouraged in future research.

### 3.5. Rodent studies

In lieu of a standardised naming convention, single-dose studies will henceforth be referred to as “acute toxicology” and repeat-dose studies will be referred to as “sub-acute toxicology”.

The Organisation for Economic Co-operation and Development (OECD) guidelines are widely acknowledged as the global standard for chemical testing. OECD guidelines are regularly updated to keep pace with scientific progress, promote best practices and align with the three principles (“3Rs”) of ethical animal testing: replacement, reduction and refinement [100]. The following OECD guidelines were used to investigate methodological quality and consistency of the included rodent studies: “*Test Guideline No. 425: Acute Oral Toxicity: Up-and-Down Procedure*” [101]; “*Test Guideline No. 407: Repeated Dose 28-Day Oral Toxicity Study in Rodents*” [102]; and “*Test Guideline No. 408: Repeated dose 90-day oral toxicity study in rodents*” [103].

For acute oral toxicity tests, the following OECD guidelines may also be considered: “*Test Guideline No. 420: Acute Oral Toxicity – Fixed Dose Procedure*” [104]; and “*Test Guideline No. 423: Acute Oral Toxicity – Acute Toxic Class Method*” [105]. Guideline No. 425 was the most recently updated guideline for acute toxicity tests, so it was given preference over guidelines 420 and 423. Additionally, Guideline No. 425 allows for dynamic dose levels, whereas guidelines 420 and 423 have fixed dose levels which are less suitable for testing compounds of unknown toxicity, such as MO seed.

#### 3.5.1. Rodent age and housing conditions.

Rodent models were the dominant means of testing MO seed toxicity, with 11 of 14 reports using rats or mice [14–19,29–31,33,36]. The preferred age of rodents in toxicological testing is 8–12 weeks old [101]. Rodent age was reported by 5 of 11 researchers: Idoko *et al.*’s [14] rats were 8–10 weeks old, Dibua *et al.*’s and Kim *et al.*’s [19,30] rats were 7–8 weeks old and Brito *et al.*’s [29] mice were 6–8 weeks old. Ezemagu *et al.* [17] stated their rats were “*age 5 weeks*” in the methodology and “*about 7 weeks old*” in the abstract. In further reading, Jackson *et al.* [106] studied the impact of rodent age on study outcomes, and concluded “*inconsistent age of rodents in many areas of scientific research may impact data quality*”. By surveying 297 respondents, they found the modal age of test rats to be 8–12 weeks and the modal age of test mice to be 6–12 weeks. These findings largely coincided with testing guidelines and with rodent age in the included reports. Historical data comparability, animal supply and animal maintenance costs were key factors affecting test rodent age. Six rodent studies did not declare rodent age [15,16,18,31,33,36]; in light of Jackson *et al.*’s [106] findings, this was considered a minor bias concern. In future toxicological research, reporting of test animal age in encouraged.

Between acquisition of test rodents and testing, an acclimatisation period of ≥ 5 d is preferred [101–103]. Acclimatisation time was reported by 6 of 11 researchers and ranged from 7–14 d [14,16,17,19,33,36]. Brito *et al.*, Ilyas *et al.* and Yurre *et al.* [15,29,31] reported rodent housing conditions but did not report acclimatisation time: acclimatisation is therefore assumed, but durations are unknown.

Preferred rodent housing conditions are displayed in Table 15 [101–103]. Rodent housing conditions were fully reported by 3 of 11 researchers [15,17,19]; rodent housing conditions were partially reported by 7 of 11 researchers [14,18,29–31,33,36]; and Ugochukwu *et al.* [16] only reported acclimatisation time. Housing temperature ranged from 19–28°C (292.15–301.15 K), exceeding the guideline range by 2–3°C (2–3 K) in two reports. In both instances, research was conducted in the tropical savanna climate of Nigeria where mean daily temperature is 25–31°C (298.15–304.15 K) year-round [14,17]. Humidity, light cycle and rodent diets adhered to OECD guidelines when reported. It was uncommon for researchers to report the number of rodents per cage: Ajayi *et al.* and Igbokwe *et al.* [33,36] reported five rodents per cage and Kim *et al.* [19] reported one rodent per cage. No research group reported same-sex housing, though test animals were commonly of the same sex within each study. Despite substantial missing data regarding rodent housing and acclimatisation, 10 of 11 researchers referenced international, national and/or institutional guidelines for ethical animal testing [14–16,18,19,29–31,33,36]. As such, there were minimal concerns of unethical or inconsistent housing of rodents, so these factors were of minimal significance to research quality and risk of bias assessments.

**Table 15:** OECD guidelines for housing conditions of rodents in toxicological studies.

Condition	Guideline value/range
Temperature	22°C (+/- 3°C) / 295.15 K (+/- 3 K)
Humidity	30–70% (50–60% during room cleaning)
Light cycle	12 h light, 12 h dark (using artificial lighting)
Diet	Conventional laboratory rodent feed and unlimited supply of drinking water
Animals per cage	Small groups (≤ 5 animals) of the same sex, or; one animal per cage if scientifically justifiable.
Acclimatisation time	≥ 5 d

#### 3.5.2. Dose administration methods.

Prior to acute studies, overnight fasting is preferred for rats and 3–4 h fasting is preferred for mice [101]; prior to sub-acute studies, fasting is not recommended for either animal [102,103]. Fasting practices were reported by 3 of 11 researchers. In adherence to guidelines, Ilyas *et al.* [15] fasted rats for 16 h prior to an acute study, but did not fast their rats prior to a sub-acute study. In contempt of guidelines, Igbokwe *et al.* [33] reported overnight fasting with mice in their acute study and with rats in their sub-acute study, and Ajayi *et al.* [36] reported a 14 h fast with rats in their sub-acute study. Inappropriate rodent fasting was considered a bias risk.

The preferred method of dose administration in toxicological studies is gavage feeding with an aqueous vehicle or incorporation of test material into rodent food or drinking water. Alternative methods are acceptable when the purpose of the study is not strictly toxicological [101–103]. Gavage or oral administration was reported by 8 of 11 researchers [14,15,17–19,30,31,33]. Ugochukwu *et al.* [16] reported oral administration in their sub-acute study and dose administration via intraperitoneal (IP) injection in their acute study; the reasoning for this choice is unclear. Brito *et al.* [29] chose IP injection because they were testing toxicity and antitumor activity of MO seed lectin. Doses were administered using an aqueous vehicle in 8 of 11 reports [14–19,31,33]. Brito *et al.* [29] paired IP injection with a phosphate-buffered saline (PBS) vehicle; PBS was also administered to the control group in this report. Dibua *et al.* [30] administered liquid MO seed oil. Ajayi *et al.* [36] did not disclose dose administration method or vehicle.

Because IP injection bypasses the test animal’s gastrointestinal tract—wherein MO seed is digested and chemically altered—studies using IP injection cannot be accurately compared to studies using gavage or oral administration. Therefore, alternate methods of MO seed administration are not recommended in toxicological testing, without solid reasoning.

#### 3.5.3. Experimental design of acute toxicity tests.

The preferred method of testing acute oral toxicity is the sequential dosing of individual animals (preferably rats), beginning at a dose one level below the estimated median lethal dose (LD50) of the substance. Dose levels should have an approximate progression factor of 3.2 and dosing should begin at 175 mg/kg bw when LD50 cannot be estimated from pre-existing literature. Consider the default OECD dose progression scheme as an example: level 1: 1.75 mg/kg bw; level 2: 5.5 mg/kg bw; level 3: 17.5 mg/kg bw; level 4: 55 mg/kg bw; level 5: 175 mg/kg bw; level 6: 550 mg/kg bw; level 7: 1750 mg/kg bw; level 8: 5000 mg/kg bw. A minimum observation period of 48 h is required after dose administration to determine survival, *moribundus* or death; observations should continue to day 14 with emphasis on delayed toxicity, delayed death and symptom recovery times. When an animal survives dosing, the test progresses to the next, higher level; when dosing results in death or *moribundus*, the test reverses to the previous, lower level. Surviving animals are retired from dosing to be observed; when testing progresses or reverses, a new animal is always selected for dosing. Testing stops when: three consecutive animals survive the highest dose (5000 mg/kg bw), or; five reversals occur in any six consecutive animals, or; four additional reversals occur at the level on which the first reversal occurred. When testing stops, LD50 is calculated following OECD guidelines [101]. Using this method, it is common to test with fewer than 10 animals.

Alternately, when pre-existing literature indicates non-toxicity, a limit test using 1–5 animals can be conducted with a dose of 2000 mg/kg bw. To satisfy regulatory needs, a limit test using 1–5 animals can also be conducted with a dose of 5000 mg/kg bw, but this is only justifiable to protect human, animal or environmental health [101]. MO seed testing aims to protect human health, so Category 5 oral dosing (5000 mg/kg bw) is justifiable. These guidelines exist to protect animal welfare, but they can also reduce project expense, labour requirements and the mental toll of animal testing. “*Test Guideline No. 420: Fixed Dose Procedure*” (5, 50, 300 and 2000 mg/kg bw) and “*Test Guideline No. 423: Acute Toxic Class Method*” (also 5, 50, 300 and 2000 mg/kg bw) can likewise be used to similar effect [104,105].

As displayed in Table 16, 7 of 11 rodent studies included an acute toxicity element. Rats were used in four studies, whereas Swiss mice were used in three studies [14–17,29,30,33]. The total number of test rodents ranged from 12–25 in six reports, suggesting inefficient experimental design when compared to contemporary guidelines [14–16,29,30,33]. MO seed dose concentrations ranged from 10–20,000 mg/kg bw in two reports, far exceeding the recommended maximum concentration (5000 mg/kg bw) [17,33]. The dose progression schemes of Idoko *et al.* and Ugochukwu *et al.* [14,16] covered three orders of magnitude and aligned relatively well with guideline values. Brito *et al.* [29] fully adhered to observation guidelines. Immediate observations were conducted for 48–72 h in three reports, partially adhering to guidelines [14,15,33]. The observation period was too short (< 48 h) in three reports [16,17,30]. Behaviour and mortality were commonly monitored, but limited additional data was gathered outside of Brito *et al.*’s [29] observations. Sub-acute toxicology was prioritised over acute toxicology in five reports, explaining limited observational variety. It was also concerning to find no negative control animals in three reports [14,16,17]. Experimental design decisions that did not adhere to OECD guidelines commonly impacted research quality judgements. Missing control groups and short clinical observation periods were also considered bias concerns.

**Table 16:** Experimental design of acute toxicity tests in rodent studies.

Report	Test organism	Total number of test organisms	Test organisms per group	Number of dosed groups	Negative control group (Y/N)	Dosing range (mg/kg bw)	Duration of observations
Brito *et al.* [29]	Swiss mice	15	5	2	Y: PBS (IP injection)	100–200 (purified lectin)	14 d
Ezemagu *et al.* [17]	Albino rats	2	2	1	N	15,000	24 h
Dibua *et al.* [30]	Wistar rats	20	5	4	Y: distilled water	1000–5000	“*overnight*”
Igbokwe *et al.* [33]	Swiss albino mice	25	5	4	Y: acacia in distilled water (2%)	5000–20,000	72 h
Idoko *et al.* [14]	Wistar rats	12	1–3	6	N	10–4800	48–72 h
Ilyas *et al.* [15]	Sprague-Dawley rats	25	5	4	Y: distilled water	600–2200	48 h
Ugochukwu *et al.* [16]	Swiss mice	17	2–3	7	N	10–1600	24 h

#### 3.5.4. Experimental design of sub-acute toxicity tests.

The preferred method of testing sub-acute oral toxicity in rodents is the concurrent, daily dosing of groups of rodents (preferably rats), at three or more dose levels, for 28 d or 90 d. OECD guidelines recommend ten rodents per dose level in 28 d studies and twenty rodents per dose level in 90 d studies, with the addition of ten or twenty rodents in the negative control group; control group animals should be dosed with the administration vehicle (e.g., distilled water) but not the test chemical. Dose levels should have an approximate progression factor of 2–4, wherein the maximum dose is estimated from pre-existing literature to cause toxic effects (but not death or severe suffering) and the minimum dose is estimated to causes no effects (i.e., NOAEL). Consider the following dose progression scheme as an example: level 1: 30 mg/kg bw/d; level 2: 100 mg/kg bw/d; level 3: 300 mg/kg bw/d; level 4: 1000 mg/kg bw/d. When pre-existing literature indicates non-toxicity at 1000 mg/kg bw/d, a three-level study may be unnecessary and a limit test may be considered [102,103].

Observational guidelines are more rigorous in sub-acute toxicity testing than in acute toxicity testing, as shown in Table 17. Additionally, a satellite group of ten rodents should be dosed in parallel with the highest dose group and observed for 14 d post-treatment to study persistence of toxic effects, recovery rates and delayed toxicity [102,103]. Using the 28 d method, it is common to test with 50–60 rodents; using the 90 d method, it is common to test with 90–110 rodents. Where acute toxicology aims to determine LD50, sub-acute toxicology aims to determine NOAEL. Consequently, animal welfare is protected in acute studies by minimising the number of test animals, whereas sub-acute studies protect animal welfare by testing at sub-lethal dose levels. Well-designed sub-acute studies do not cause severe animal suffering or mortality.

**Table 17:** OECD guidelines for observations in sub-acute oral toxicity studies in rodents.

Observation type	Observation frequency: 28 d study	Observation frequency: 90 d study
Morbidity and mortality	Twice a day	Twice a day
“*General clinical observations*”	Once a day	Once a day
“*Detailed clinical observations*”	Once a week	Once a week
Food consumption rate	Once a week	Once a week
Body weight change	Once a week	Once a week
Sensory reactivity	Week 4	≥ Week 11
Ophthalmological examination	N/A	Before first dose and end of study
Haematology	End of study	End of study
Clinical biochemistry (e.g., renal panel, hepatic panel)	End of study	End of study
Gross necropsy	End of study	End of study
Histopathology	End of study	End of study
Reproductive organ pathology	N/A	End of study

As displayed in Table 18, 9 of 11 rodent studies included a sub-acute toxicology element [14–19,31,33,36]. Test duration was commonly 21–34 d, although two studies were significantly shorter at 9 d and 14 d [17,19]. A modal duration of 28 d suggested some adherence to “*Test Guideline No. 407: Repeated Dose 28-Day Oral Toxicity Study*” or similar methodologies [102]. In seven studies, the total number of rodents ranged from 8–30, with 4–5 rodents per dose level [14–18,33,36]. This was fewer than recommended for 28 d studies (50–60 rodents total; 10 rodents per group). Kim *et al.* [19] adhered to guidelines for this variable, testing with 50 rats across five groups of ten. The number of mice used by Yurre *et al.* [31] was unclear. Dose concentration commonly ranged from 50–6400 mg/kg bw/d, exceeding the recommended maximum concentration (1000 mg/kg bw/d) in three reports [15,17,19]. Dose progression factor was commonly 2–3, with three or more dose levels. Exceptions to this were Idoko *et al.* and Yurre *et al.* [14,31] who tested with one dose level, and Ugochukwu *et al.* [16] who tested with two dose levels. Negative control rodents were included in all experiments, and they were commonly dosed with distilled water. Mortality was universally observed. Renal and hepatic biochemistry, haematology, histology and body weight change were each observed in 6 of 9 reports. Specialist observations were made in two studies and included indices of reproductive pathology and cardiac pathology [18,31]. In comparison to acute toxicology studies, observations made in sub-acute studies were generally more rigorous and standardised. Non-compliant experimental design again impacted research quality judgements; short clinical observation periods were also considered a bias concern.

**Table 18:** Experimental design of sub-acute toxicity tests in rodent studies.

Report	Test organism	Total number of test organisms	Test organisms per group	Number of dosed groups	Negative control group (Y/N)	Dosing range (mg/kg bw/d)	Duration of study
Ezemagu *et al.* [17]	Albino rats	32	4	7	Y: distilled water	100–6400	9 d
Attah *et al.* [18]	Wistar rats	30	5	5	Y: distilled water	50–500 (plus *ad libitum* group)	34 d
Yurre *et al.* [31]	C57BL/6 mice	Unclear	≤ 14	1	Y: ultrapure water	5 (purified lectin)	21 d
Igbokwe *et al.* [33]	Wistar rats	20	5	3	Y: acacia in distilled water (2%)	100–500	30 d
Kim *et al.* [19]	Sprague-Dawley rats	50	10	4	Y: distilled water	78–2571	14 d
Idoko *et al.* [14]	Wistar rats	8	4	1	Y: distilled water	480	28 d
Ilyas *et al.* [15]	Sprague-Dawley rats	20	5	3	Y: distilled water	600–1800	21 d
Ajayi *et al.* [36]	Wistar rats	25	5	4	Y: distilled water	100–1000	28 d
Ugochukwu *et al.* [16]	Wistar rats	15	5	2	Y: normal saline	100–200	28 d

#### 3.5.5. Results of acute toxicity tests.

Researchers who exposed MO seed powder to warm water (> 30°C / 303.15 K) during defatting or secondary processing (i.e., hot preparation: inactivated myrosinase/no isothiocyanate or nitrile production) reported few toxic effects in acute studies. Testing an aqueous extract with rats, Ilyas *et al.* [15] reported no mortality at 600–2200 mg/kg bw, reversible dullness at 1800–2200 mg/kg bw and no other significant effects; as such, they did not determine a LD50 value. Testing defatted MO seed powder with rats, Idoko *et al.* [14] reported no mortality at 10–4800 mg/kg bw and no other significant effects; similarly, they did not determine a LD50 value. MO seed was mixed with warm water (~38–43°C / 311.15–316.15 K) in both studies, suggesting a low inactivation temperature for MO-derived myrosinase.

Researchers who did not expose MO seed powder to water or elevated temperatures (> 30°C / 303.15 K) prior to the aqueous vehicle (i.e., cold–dry preparation until administration: active myrosinase/possible *in vivo* isothiocyanate and nitrile production) reported more severe toxic effects. Testing an ethanol extract with mice, Igbokwe *et al.* [33] reported mortality rates of 20% at 10,000 mg/kg bw and 100% at 15,000–20,000 mg/kg bw; an LD50 value of 14,000 mg/kg bw was calculated. They also reported changes in behaviour and psychomotor reflexes at 15,000–20,000 mg/kg bw. Testing MO seed powder with two rats, Ezemagu *et al.* [17] reported a 50% mortality rate at 15,000 mg/kg bw, alongside significant decreases in blood urea and creatinine. Renal histology displayed tubular atrophy and necrosis of the glomeruli. Due to inconsistencies in dose range and dose progression factor, it was unclear whether these findings aligned with the theory of temperature-dependant toxicity.

Two research groups administered MO seed via IP injection in acute studies. Resultantly, phytochemicals bypassed digestion by the gastrointestinal tract and caused toxicity at lower concentrations. Testing an ethanol extract with mice, Ugochukwu *et al.* [16] reported mortality rates of 50% at 400 mg/kg bw, 66.7% at 1000 mg/kg bw and 100% at 800 mg/kg bw and 1600 mg/kg bw; a LD50 value of 282.84 mg/kg bw was calculated. Testing purified MO seed lectin with mice, Brito *et al.* [29] reported a 60% mortality rate at 200 mg/kg bw alongside depressive symptoms, drowsiness, constipation and abdominal spasms. At 100 mg/kg bw, they reported similar symptoms and significant decreases in food consumption rate, water consumption rate and weight gain in the 14 d observation period. These findings suggest antinutritional properties in MO seed lectin and a LD50 value of 100–200 mg/kg bw when administered intraperitoneally. To reiterate, studies using IP injection cannot be directly compared to oral toxicity tests.

Dibua *et al.* [30] were the only researchers to test an n-hexane lipid extract of MO seed. They reported agitation, unstable movement, behavioural perturbations, writhing, sedation and diarrhoea in rats dosed at 1000–5000 mg/kg bw. Most rats recovered from these symptoms in 9–14 h, but restlessness continued for an undisclosed duration in rats dosed at 2900–5000 mg/kg bw. No mortality was reported at any dose, so a LD50 value was not determined. The dose range of Idoko *et al.* [14]—the only researchers to test defatted MO seed—is consistent with the dose range of Dibua *et al.* [30], yet toxicity was only reported by the latter. These finding implicate the MO seed lipids as a toxic fraction. Due to the small sample size, this theory requires additional testing.

Among acute oral toxicity tests, MO seed was generally concluded to be “non-toxic” or “safe”. These conclusions were made in response to high oral LD50 values (> 5000 mg/kg bw), reversible toxicities at high dose concentrations (≥ 1000 mg/kg bw) or absence of mortality and toxicity within chosen dose ranges. In comparison to contemporary testing guidelines, the experimental design of acute toxicity tests more closely resembled that of sub-acute toxicology guidelines. Additionally, post-administration observation periods were commonly too short, negative control groups were not universally used and methods of MO seed processing were highly variable. Data regarding safety limits from acute toxicity tests are tabulated in Table 19.

**Table 19:** LD50 data from acute toxicity tests with *M. oleifera* seed in rodent studies.

Report	MO seed product	Maximum processing temperature	MO seed dosing range (mg/kg bw)	Lethal dose(s) (mg/kg bw)	LD50 (mg/kg bw)
Brito *et al.* [29]	Purified lectin	28°C (301.15 K)	100–200	200	100 – 200^a^
Ezemagu *et al.* [17]	Seed powder	Room temperature^b^	15,000	15,000	15,000^c^
Dibua *et al.* [30]	n-hexane lipid extract	Room temperature	1000–5000	(no mortality)	N/A^d^
Igbokwe *et al.* [33]	Ethanol extract	Room temperature^b^	5000–20,000	10,000–20,000	14,000^c^
Idoko *et al.* [14]	Defatted seed powder	“*warm water*”; 40°C (313.15 K) baking	10–4800	(no mortality)	N/A^d^
Ilyas *et al.* [15]	Aqueous extract	“*warm water*” used in extraction	600–2200	(no mortality)	N/A^d^
Ugochukwu *et al.* [16]	Ethanol extract	Room temperature	10–1600	400–1600	282.84^a^

^a^MO seed dose administered via IP injection.

^b^Room temperature assumed in lieu of precise temperature data.

^c^MO seed dose administered orally or via oral gavage.

^d^LD50 value cannot be determined in absence of lethal dose data.

LD50 data is cross-referenced with judgements from conflict of interest, research quality and risk of bias assessments in Table 20. Brito *et al.*’s [29] report received “highest quality” and “low risk of bias” judgements, indicating reliable research and a trustworthy LD50 value for IP administration of MO seed lectin. Unfortunately, tertiary processing of MO seeds and administration via IP injection limit the utility of this safety limit: when used as a dietary supplement, MO seeds are typically eaten whole. IP injection was also used to determine Ugochukwu *et al.*’s [16] safety limit; this report received “lowest quality” and “high risk of bias” judgements, so the data is less reliable. Ezemagu *et al.* [17] likewise received “lowest quality” and “high risk of bias” judgements. Bias risk in this report was associated with experimental design: the acute toxicology test did not include a negative control group, study duration fell short of OECD guidelines, rodent fasting practices were not reported, there was a discrepancy in the reporting of rodent age and there was no reference to external procedures or ethical guidelines for animal testing. Ezemagu *et al.* [17] were the only researchers to report direct evidence of kidney injury in acute toxicology testing, in the form of a photomicrograph cross-section showing severe tubular atrophy and necrosis of the glomeruli. Due to the extreme dose (15,000 mg/kg bw) and bias risk, this finding cannot be considered reliable evidence of nephrotoxicity in MO seeds. Igbokwe *et al.’*s [33] research was marginally more reliable, receiving “lowest quality” and “some concerns” judgements. Bias concerns were associated with experimental design and missing data: rodents were subject to inappropriate fasting prior to the acute toxicology test and graphical data was reported without standard deviation, obscuring the significance of some results. Overall, the LD50 values examined in this systematic review were non-applicable to human consumption of MO seeds and/or of low reliability.

**Table 20:** LD50 data and judgements from conflict of interest, research quality and risk of bias assessments.

Report	LD50 (mg/kg bw)	Conflict of interest judgement	Overall research quality judgement	Overall risk of bias judgement
Brito et al. [29]	100 – 200^a^	No notable concern about conflict of interest	Highest quality	Low risk of bias
Ezemagu et al. [17]	15,000^b^	No notable concern about conflict of interest	Lowest quality	High risk of bias
Igbokwe et al. [33]	14,000^b^	No notable concern about conflict of interest	Lowest quality	Some concerns
Ugochukwu et al. [16]	282.84^a^	No notable concern about conflict of interest	Lowest quality	High risk of bias

^a^MO seed dose administered via IP injection.

^b^MO seed dose administered orally or via oral gavage.

#### 3.5.6. Results of sub-acute toxicity tests.

Researchers who exposed MO seed powder to warm water (> 30°C / 303.15 K) during defatting or secondary processing (i.e., hot preparation: inactivated myrosinase/no isothiocyanate or nitrile production) again reported few toxic effects. Testing aqueous extracts with rats for 21 d, Ilyas *et al.* [15] reported no significant changes in haematological or biochemical indices at 600–1800 mg/kg bw/d. However, they did report dose-dependent decreases in body weight gain at all dose levels (600–1800 mg/kg bw/d); the NOAEL was therefore indeterminable. Testing defatted MO seed powders with rats for 28 d, Idoko *et al.* [14] reported no mortality, clinical observations, haematological changes nor histological changes at 480 mg/kg bw/d (the only dose level tested). By day 28, test animals weighed significantly less than control animals, blood creatinine was significantly decreased and blood phosphate was significantly increased; these findings were attributed to antinutrient phytochemicals, but a NOAEL was indeterminable. Idoko *et al.* [14] also attributed the relative safety of their MO seeds to defatting, whereas heat inactivation of the MOB reaction is the theoretical cause proposed by this review. Among the sub-acute studies, Idoko *et al.* and Ilyas *et al.* [14,15] were the only research groups to report decreased weight gain in rodents dosed with MO seed. While the sample size is small, it is theorised that glucosinolates—preserved by heat inactivation of myrosinase—possess antinutrient and/or appetite-suppressing properties. In a 2022 review on antinutrient phytochemicals, glucosinolates were implicated, though further research is required to verify antinutrient activity in glucomoringin and glucosoonjnain [107].

Researchers who exposed MO seed powder to water (< 30°C / 303.15 K) during secondary processing (i.e., cold–wet preparation: active myrosinase/*in vitro* isothiocyanate production) reported more severe toxic effects. Testing aqueous–ethanol extracts with rats for 14 d, Kim *et al.* [19] reported an 80% mortality rate at 2571 mg/kg bw/d, with mortality beginning on day 2 of testing. Irregular respiration and respiratory crackling were observed at 257–2571 mg/kg bw/d, and piloerection was observed (more commonly in male rats) at 772–2571 mg/kg bw/d. A significant increase in white blood cell counts, due to increased neutrophil concentration, was observed in surviving female rats at 2571 mg/kg bw/d. Surviving females were also found to have increased liver-to-body weight ratios, decreased thymus size and decreased lymphocyte counts in the thymus and spleen at 772–2571 mg/kg bw/d. Gross necropsy in non-surviving animals (2571 mg/kd bw/d) revealed decreased thymus and spleen sizes, decreased spleen lymphocyte count, gastrointestinal distention, discolouration of the stomach and degeneration of testicular germinal cells and epididymis cells. Kim *et al.* [19] concluded their report with a NOAEL of 257 mg/kg bw/d for MO seed aqueous–ethanol extract, standardised to 100 mg/kg bw/d for MO seed isothiocyanates. Testing aqueous–methanol extracts with rats for 34 d, Attah *et al.* [18] reported collapse and necrosis of the renal tubules at all dose levels (50–500 mg/kg bw/d) in dams and their offspring; the NOAEL of their extract was therefore indeterminable. Poor liver architecture was reported at 300–500 mg/kg bw/d in dams and at 50 and 500 mg/kg bw/d in pups. In dams dosed at 500 mg/kg bw/d pup gestation lengths were significantly decreased; in dams dosed at 50–100 mg/kg bw/d pup birth weights were significantly decreased. Female pups also showed evidence of intra-uterine growth restriction and masculinisation. Following these findings, Attah *et al.* [18] administered a single dose of defatted aqueous-methanol extract at 300 mg/kg bw. No histological effects were observed, leading researchers to attribute toxicity to the lipid fraction. While this conclusion aligns with the conclusion of Idoko *et al.* [14], a single dose cannot be accurately compared to a 35-day repeat-dose. Moreover, the procedure for this test was not disclosed, so it is unclear how many rodents were tested and for how long they were observed before sacrifice.

Researchers who did not expose MO seed powder to water or elevated temperatures (> 30°C / 303.15 K) prior to the aqueous vehicle (i.e., cold–dry preparation until administration: active myrosinase/possible *in vivo* isothiocyanate and nitrile production) reported toxic effects at lower concentrations than the previously discussed studies. Testing methanol extracts with rats for 28 d, Ajayi *et al.* [36] reported severe renal toxicity at 1000 mg/kg bw/d, possible hepatic toxicity at 200–1000 mg/kg bw/d and significant changes to renal and hepatic biochemistry at all dose levels (100–1000 mg/kg bw/d); the NOAEL was indeterminable. They also reported agitation, confusion, disorientation, tremors at 1000 mg/kg bw/d, significantly decreased packed cell volume at 200 mg/kg bw/d and significantly reduced haemoglobin at 100 mg/kg bw/d. Toxicity was attributed to isothiocyanate content in this study. Testing ethanol extracts with rats for 30 d, Igbokwe *et al.* [33] reported significant changes in biochemistry and haematology at all dose levels (100–500 mg/kg bw/d); the NOAEL was again indeterminable. Histological findings indicated renal degeneration at 250 mg/kd bw/d and potential hepatic degeneration at 100–250 mg/kg bw/d. Increased weight gain was also reported at all dose levels when compared to control animals. Testing MO seed powders with rats for 9 d, Ezemagu *et al.* [17] reported a mortality rate of 25% at 6400 mg/kg bw/d and indications of renal necrosis at 200–6400 mg/kg bw/d. Significant changes to renal biochemistry began at 200 mg/kg bw/d, but were most prevalent at 1600–6400 mg/kg bw/d. The NOAEL was determined to be 100 mg/kg bw/d. Testing ethanol extracts with rats for 28 d, Ugochukwu *et al.* [16] reported a significantly increased platelet count at 200 mg/kg bw/d as the only finding of their sub-acute study. Hepatic biochemistry findings were considered insignificant, despite decreases in AST from “170.20 ± 48.8” to “69.00 ± 4.09”; this may be an oversight or a typographical error. No observable adverse effects were detected at their lowest dose level (100 mg/kg bw/d).

By testing purified MO seed lectin with mice for 21 d, Yurre *et al.* [31] were the only researchers to conduct sub-acute toxicology with a tertiary extract. Focussing exclusively on cardiac effects, they observed no significant changes to any index of toxicity at 5 mg/kg bw/d (the only dose level tested).

Of the nine sub-acute studies included in this review: three tested a dose level low enough to determine a NOAEL [16,17,19]; two tested a single dose level and were unable to determine a NOAEL as a result [14,31]; and four studies reported adverse effects at all dose levels [15,18,33,36]. A NOAEL of 100–257 mg/kg bw/d for MO seed powders and secondary extracts appeared to be the consensus safety limit, although the sample size was small (n = 3) [16,17,19]. Attah *et al.* [18] reported renal and reproductive toxicity at 50 mg/kg bw/d, bringing into question this NOAEL range and the common dose range, which rarely fell below 100 mg/kg bw/d. Despite these findings, four reports with sub-acute toxicology elements promoted MO seed safety for dietary and/or medicinal use in their abstracts [14–16,36]. Data regarding safety limits from sub-acute toxicity tests are tabulated in Table 21.

**Table 21:** NOAEL data from sub-acute toxicity tests with *M. oleifera* seed in rodent studies.

Report	MO seed product	Maximum processing temperature	MO seed dosing range (mg/kg bw/d)	Lethal dose (mg/kg bw/d)	Toxic dose(s) (mg/kg bw/d)	NOAEL (mg/kg bw/d)
Ezemagu *et al.* [17]	Seed powder	Room temperature^a^	100–6400	6400	200 - 6400	100^b^
Attah *et al.* [18]	Aqueous–methanol extract (1:1)	Room temperature^a^	50–500	(no mortality)	50–500	N/A^C^
Yurre *et al.* [31]	Purified lectin	28°C (301.15 K)	5	(no mortality)	(no toxicity)	N/A^d^
Igbokwe *et al.* [33]	Ethanol extract	Room temperature^a^	100–500	(no mortality)	100–500	N/A^C^
Kim *et al.* [19]	Aqueous–ethanol extract	37°C (310.15 K) following aqueous extraction	78–2571	2571	257–2571	257^b^
Idoko *et al.* [14]	Defatted seed powder	“*warm water*”; 40°C (313.15 K) baking	480	(no mortality)	480	N/A^C^
Ilyas *et al.* [15]	Aqueous extract	“*warm water*” used in extraction	600–1800	(no mortality)	600–1800	N/A^C^
Ajayi *et al.* [36]	Methanol extract	“*cold extraction*”	100–1000	(no mortality)	100–1000	N/A^C^
Ugochukwu *et al.* [16]	Ethanol extract	Room temperature	100–200	(no mortality)	200	100^b^

^a^Room temperature assumed in lieu of precise temperature data.

^b^MO seed dose administered orally or via oral gavage.

^c^NOAEL value cannot be determined when toxicity occurred at all dose concentrations.

^d^NOAEL value cannot be determined in absence of toxicity data.

NOAEL data is cross-referenced with judgements from conflict of interest, research quality and risk of bias assessments in Table 22. All reports that produced NOAEL data received “high risk of bias” judgements. Ezemagu *et al.* [17] received a “lowest quality” judgement in addition to the bias judgement; bias risk was associated with experimental design, as discussed in Section 3.5.5. Ugochukwu *et al.* [16] likewise received a “lowest quality” judgement. Bias risk in sub-acute tests was associated with missing data and selective reporting of data: biochemical data (renal panel) and histopathological data were not generated or not reported, and conclusions were drawn in omission of reported data, as discussed in Section 2.2. Kim *et al.* [19] declared financial conflicts of interest and received a “higher quality” judgement for their research. Bias risk was again associated with missing data and selective reporting of data: biochemical data (hepatic panel and renal panel) were not generated or not reported, and conclusions were again drawn in omission of reported data, as discussed in Section 2.2. Significant biochemical and histopathological changes were reported by other researchers at MO seed doses of 100–1000 mg/kg bw/d [33,36]. Therefore, the NOAEL values of Ugochukwu *et al.* [16] and Kim *et al.* [19] were determined in omission of key toxicological parameters. Similarly, Ezemagu *et al.* [17] did not report hepatic data for biochemistry or histopathology, but the scope of their study was intentionally limited to renal toxicity. Overall, NOAEL values examined in this systematic review were of low reliability. The authors of this review suggest cessation of MO seed promotion and consumption until safety limits can be more reliably tested, preferably in adherence to testing guidelines. Policy makers must be cautious when assessing contemporary literature: abstract and concluding statements may not reflect reported data, and reported data may be missing key toxicological elements.

**Table 22:** NOAEL data and judgements from conflict of interest, research quality and risk of bias assessments.

Report	NOAEL (mg/kg bw/d)	Conflict of interest judgement	Overall research quality judgement	Overall risk of bias judgement
Ezemagu *et al.* [17]	100^a^	No notable concern about conflict of interest	Lowest quality	High risk of bias
Kim *et al.* [19]	257^a^	Notable concern about conflict of interest	Higher quality	High risk of bias
Ugochukwu *et al.* [16]	100^a^	No notable concern about conflict of interest	Lowest quality	High risk of bias

^a^MO seed dose administered orally or via oral gavage.

Indications of kidney injury were reported in four sub-acute toxicology tests that included renal biochemistry and/or renal histopathology [17,18,33,36]. Two reports that included renal elements reported no indication of kidney injury [14,15]; three reports did not include renal elements [16,19,31]. Researchers who detected renal injury all reported histopathological evidence, though this evidence was inconsistent: Attah *et al.* reported necrosis of the renal tubules in rat dams, beginning at 50 mg/kg bw/d [18]; Igbokwe *et al.* reported degeneration of renal tissues at 250 mg/kg bw/d [33]; Ezemagu *et al.* reported necrotic glomeruli, beginning at 400 mg/kg bw/d [17]; Ajayi *et al.* reported severe necrosis of the renal tubules at 1000 mg/kg bw/d [36]. Findings from renal biochemistry were similarly inconsistent: Igbokwe *et al.* reported significant increases in serum creatinine at all doses, indicating kidney injury and supporting their histopathological evidence [33]; Ezemagu *et al.* and Ajayi *et al.* both reported significant decreases in serum creatinine and serum urea, so their biochemical evidence did not support their histopathological evidence [17,36]. Ultimately, all studies that included renal elements were deemed to have “lower” or “lowest” research quality. Therefore, the question of whether MO seeds are contributing to AKI incidence cannot be answered on account of inconsistent evidence, limited sample size and low research quality. Data pertaining to kidney injury is displayed in Table 23.

**Table 23:** Renal biochemistry and renal histopathology data from sub-acute toxicity tests with *M. oleifera* seed in rodent studies.

Report	MO seed product	Evidence from renal biochemistry	Evidence from renal histopathology
Ezemagu *et al.* [17]	Seed powder	1600 - 6400 mg/kg bw/d: significant decrease in serum creatinine^a^; 200, 1600 & 6400 mg/kg bw/d: significant decrease in serum urea^a^	200 mg/kg bw/d: distortion of renal tissue; 400 - 6400 mg/kg bw/d: necrosis of renal tubules and glomeruli
Attah *et al.* [18]	Aqueous–methanol extract (1:1)	N/A^b^	50–500 mg/kg bw/d: necrosis of renal tubules (rat dams only); 300–500 mg/kg bw/d: necrosis of renal tubules (rat pups only)
Yurre *et al.* [31]	Purified lectin	N/A	N/A
Igbokwe *et al.* [33]	Ethanol extract	100–500 mg/kg bw/d: significant increase in serum creatinine	200 mg/kg bw/d: “*indications of kidney degeneration*”
Kim *et al.* [19]	Aqueous–ethanol extract	N/A	No renal findings reported (histopathology images only provided for spleen and testis tissues)
Idoko *et al.* [14]	Defatted seed powder	480 mg/kg bw/d: significant decrease in serum creatinine^a^	No gross or microscopic lesions observed in kidney tissue cross-sections
Ilyas *et al.* [15]	Aqueous extract	No significant biochemical changes	N/A
Ajayi *et al.* [36]	Methanol extract	100–1000 mg/kg bw/d: significantly decreased serum creatinine^a^; 100 mg/kg bw/d: significantly decreased blood urea nitrogen^a^	1000 mg/kg bw/d: “*renal tubules are severely necrotic and shrunken*”
Ugochukwu *et al.* [16]	Ethanol extract	N/A	N/A

^a^Significant decreases in serum creatinine or serum urea are not evidence of kidney injury.

^b^“N/A” indicates no testing took place.

### 3.6. Non-rodent studies

Toxicity tests were conducted with non-rodent organisms in 3 of 14 reports, as shown in Table 24. To accommodate alternate test organisms and alternate aims, the experimental design of these tests differed significantly from rodent studies. Because they did not pertain to the dietary or medicinal safety of MO seeds, these reports are discussed in less detail and with less scrutiny than rodent studies.

**Table 24:** Experimental design of non-rodent studies.

Report	Test organism	Total number of test organisms	Test organisms per group	Number of dosed groups	Negative control group	Dosing range	Number of doses/duration of study
Potestà *et al.* [32]	Human cells (THP-1, Kurkat, PBMCs)	N/A	N/A	8	Y: water	0.01–10 mg/ml	Single dose; 72 h exposure
Santana Silva *et al.* [35]	Zebrafish (*Danio rerio*)	144 embryos	24	5	Y: water	12.5–200 mg/L (purified lectin in water)	Daily repeat-dose; 4 d
Chinenyenwa & Godson [34]	Mosquito larvae (*Anopheles gambiae*) & guppies (*Poecilia reticulata*)	Mosquito larvae: 120; guppies: unclear	Mosquito larvae: 20; guppies: 20	Mosquito larvae: 5; guppies: 3	Y: distilled water	Mosquito larvae: 1.16–8.7 mg/L; guppies: 10–30 mg/L	Single dose followed by observation period. Mosquito larvae: 5 d; guppies: 24 h

Potestà *et al.* [32] were the only researchers to test with human cells (THP-1, Kurkat and peripheral blood mononuclear cells). This study focussed on the anti-proliferative, cytotoxic and pro-apoptotic properties of MO leaves and seeds, but also included EC50 and LD50 data for MO seed extracts. In this trial, two aqueous extracts of MO seed were prepared: one with boiling water (hot preparation) and one with freezing water (cold preparation). Hot preparations exhibited weaker cytotoxic effects than cold preparations, returning higher EC50 and LD50 concentrations in all instances within the measurable range (≤ 10 mg/ml). While not discussed in their publication, it can be inferred that boiling water inactivated myrosinase, occluding the MOB reaction and isothiocyanate-induced toxicity. In the cold preparation, myrosinase was preserved and the MOB reaction produced toxic isothiocyanates in proximity to the human cells. These findings complement the theory of temperature-dependent toxicity explored in Section 3.1.1 and Sections 3.5.5–3.5.6.

Two reports evaluated the bio-insecticidal capacity of MO seeds. As a two-part study, Coelho *et al.* and Santana Silva *et al.* [35,98] tested purified MO seed lectin on *Aedes aegypti* larvae (yellow fever mosquito) and *Danio rerio* larvae and embryos (zebrafish), whereas Chinenyenwa & Godson [34] tested a MO seed aqueous extract on *Anopheles gambiae s.s.* larvae (mosquito) and *Poecilia reticulate* (guppy fish). Guppy fish and zebrafish naturally predate mosquito eggs and larvae, hence their use in mosquito control strategies; they are also used to evaluate ecotoxicity. Following 96 h of exposure, Santana Silva *et al.* [35] reported median lethal concentration (LC50) values of 49 mg/L for zebrafish embryos, 135 mg/L for zebrafish larvae and 197 mg/L for mosquito larvae. As such, they did not recommend MO seed lectin as a mosquito larvicide in the presence of non-target organisms [98]. Follow 24 h of exposure, Chinenyenwa & Godson [34] reported LC50 values of 36.4 mg/L for guppies and 2.51 mg/L for mosquito larvae. In contrast with the first study, they went on to recommend Moringa-based larvicides for mosquito control. Between the two reports, LC50 values for mosquito larvae differed by approximately two orders of magnitude; this is likely the result of disparate MO seed processing methods or exposure times.

### 3.7. Data analysis

Methods of data analysis were disclosed in 13 of 14 toxicology reports. Mean ± standard deviation was universally used and ANOVA was used in nine instances. Additional tests included Abbott’s formula (n = 2), Bartlett’s (n = 1), Bonferroni correction (n = 2), chi-squared (n = 1), Duncan's multiple-range (n = 1), Dunn’s, Dunnett’s (n = 2), Kolmogorov–Smirnov (n = 2), Kruskal-Wallis (n = 1), Levene’s (n = 1), Mann–Whitney U (n = 1), Probit regression (n = 2), Student’s t-test (n = 5), Tukey’s (n = 3) and least significant difference (n = 1). Where disclosed, a confidence level of 95% was used [14–19,29,31–36]. Dibua *et al.* [30] did not report analytical procedures, impacting research quality as a result.

### 3.8. Dietary safety and water treatment with *M. oleifera* seeds

In sub-acute rodent studies, the minimum concentrations at which toxicity was reported was 50 mg/kg bw/d. Assuming rodent models are representative of human toxicology, a 62 kg human (the global average body mass) may begin to experience toxicity attributable to MO seed consumption at 3100 mg/d [108]. A single seed weighs ~300 mg, so this equates to ~10 seeds per day [109,110]. The Association of UK Dietitians defines a portion of edible, unsalted nuts (e.g., peanuts, cashew nuts) as 30,000 mg/d [111]. Therefore, it is unlikely that MO seeds can be incorporated into the human diet as a substitute or analogue for commonly eaten nuts without risking toxicity via overconsumption.

When using MO seeds as a coagulant in water treatment, coagulant dosing concentrations typically range from 5–350 mg/L. Assuming a worst-case scenario where CFS consistently fails and little gravitational settling occurs (i.e., due to incorrect operational parameters and electrostatic suspension of coagulant), MO seed concentrations in treated water can be assumed to be ≤ 350 mg/L. It is recommended that an adult drinks ~3 L of water per day [112]. With this value in mind, ≤ 1050 mg/d of MO seed coagulant can be ingested via drinking water in a persistent worse-case scenario. This dose is approximately three times lower than the lowest theoretical safety limit of 3100 mg/d for a 62 kg adult. In a scenario where CFS fails due to a consistent 10X dosing miscalculation, MO seed coagulant may be ingested at a rate of ≤ 10,500 mg/d and toxicity may occur. It should also be noted that pregnant and breastfeeding women require ~5 L of water per day to remain hydrated, so their coagulant intake can be ~ 60% higher than other adults [112].

Barring worst-case scenarios, drinking water treated with MO seed is unlikely to cause toxicity as a lone source of MO phytochemicals. However, it is likely that additional MO products—derived from the leaves, seeds, seed pods, bark and roots of the plant—are used simultaneously wherever the tree is grown. A growing number of publications are identifying nephrotoxic herbal products, and 35% of acute renal injuries in Africa are believed to be caused by them [113–116]. Dietary and medicinal uses of MO must be considered when promoting the adoption of MO seed water treatment.

### 3.9. Allergy

Seed storage proteins, such as 2S albumins, 11S globulins and 12S globulins, are known allergens found in Brassicale species and other plant seeds. Napin (a 2S albumin) and cruciferin (a 11S globulin) are the two most common seed storage proteins in mustard and rapeseed [117]. *Mo*-CBP3 is also a 2S albumin, and its four known isoforms share amino acid sequences with napin and several other allergenic 2S albumins (35–45% identity on an 80 amino acid window). Cross-reaction in individuals sensitised to Brassicale allergens is therefore possible, and recommendations for further testing have been made [118].

Theory aside, the consumption of MO seeds and leaves has already resulted in anaphylaxis in a number of case studies [119–121]. Additionally, occupational asthma has been reported in a factory technician tasked with processing MO seeds [122]. The rapid adoption of MO products will continue to cause sensitisation events in producers and consumers. To prevent this outcome, dust masks should be worn whenever dusts are generated. Ichrak [120] also recommends the addition of allergy warning labels to products containing MO materials.

Allergy was rarely mentioned in the included reports, but it is worth consideration in context of MO seed water treatment. Allergenic potential cannot be reduced via heating because MO seed allergens are heat-stable to > 100°C (373.15 K) and MOCPs lose their CFS capacity at ~90°C (363.15 K) [123–126]. Additionally, it may not be possible to separate MOCPs and allergens using solubility fractionation (i.e., secondary processing) due to their reported similarities. Because of the expensive and complexity of tertiary processing, the routine purification of MOCPs from allergens will also be unsustainable for developing regions. Moreover, food allergies are primarily prevented by avoidance. Drinking water cannot be easily avoided, and MO-contaminated water could drive sensitised individuals to unsafe or unaffordable water sources.

In individuals sensitised to peanut protein, systemic reactions can occur following a 5 mg dose [127]. MO seeds contain ~40% protein, and processing methods aim to concentrate the protein fraction to improve CFS potential [128]. Assuming the standard coagulant dosing range of 5–350 mg/L, water treatment with MO seed powder introduces ~2–140 mg of protein per litre of water. If MO seed proteins cause systemic allergenicity at a similar concentration to peanut proteins, drinking water treated with MO seeds may be unsafe for sensitised individuals even if residual coagulant persists at low concentrations. To minimise allergenic potential, water treatment methodologies must be optimised to reduce residual coagulant.

## 4. Summary of findings

- The reliability of contemporary toxicological research with MO seed was low. Financial conflicts of interest were detected in one report. Research quality assessments returned five “highest quality” or “higher quality” judgments and nine “lower quality” or “lowest quality” judgements. Risk of bias assessments returned six “low risk of bias” judgements, two “some concerns” judgements, and six “high risk of bias” judgements. Reports with “high risk of bias” judgements were commonly found to underestimate MO seed toxicity via selective reporting of data.
- MO seeds were acquired locally in 12 of 14 reports, demonstration the wide availability of the tree. Species was verified by taxonomist or herbarium in 11 of 14 reports. The age of MO seeds at the time of testing was only declared in one report, and seed storage conditions were only declared in three reports. As a result, phytochemical variability cannot be ruled out, despite species verification.
- Methods of MO seed processing were highly inconsistent. Primary-processed MO seed powders were tested in 2 of 14 reports, secondary-processed MO seed extracts were tested in 9 of 14 reports and tertiary-processed MO seed lectin was tested in 3 of 14 reports. The following solvents were used for secondary processing: aqueous (n = 3), ethanol (n = 2), aqueous-ethanol (n = 1), methanol (n = 1), aqueous-methanol (n = 1) and n-hexane (n = 1).
- Phytochemical profiles of MO seed preparations were altered by exposure to water and elevated temperatures (> 30°C / 303.15 K) during processing. Hot preparations (i.e., inactivated myrosinase/no isothiocyanate or nitrile production) were carried out in 3 of 11 reports, cold–wet preparations (i.e., active myrosinase/*in vitro* isothiocyanate production) were carried out in 8 of 11 reports, and cold–dry preparations (i.e., active myrosinase/*in vivo* isothiocyanate and nitrile production) were carried out in 4 of 11 reports. Preparation types were further complicated by the aqueous dosing vehicle in rodent studies.
- Rodents were used to study toxicity in 11 of 14 reports. Acute toxicology (single dose) was tested in 7 of 11 rodent studies and sub-acute toxicology (repeat-dose) was tested in 9 of 11 rodent studies.
- Experimental designs of acute toxicology studies were generally inefficient and unreliable by contemporary standards. Observation periods were too short in 3 of 7 reports and there were no negative control animals in 3 of 7 reports. Moreover, the maximum recommended dose (5000 mg/kg bw) was exceeded in two reports, suggesting unnecessary animal suffering.
- Experimental designs of sub-acute toxicology studies more closely adhered to contemporary guidelines, and all reports included negative control animals. Test groups contained too few rodents (≤ 5) in 7 of 9 reports and the post-test 14 day observation period was commonly omitted. The maximum recommended dose (1000 mg/kg bw/d) was exceeded in three reports, again suggesting unnecessary animal suffering.
- Two acute oral toxicity tests returned LD50 values of 14,000–15,000 mg/kg bw. Two acute toxicity tests that administered MO seed via IP injection returned LD50 values of 100–283 mg/kg bw. The reliability of LD50 data was low. Two acute oral toxicity tests returned no mortality and limited toxic effects; researchers were unable to determine LD50 in these instances.
- Three sub-acute oral toxicity tests returned NOAEL values of 100–257 mg/kg bw/d, although renal and reproductive toxicity was also reported at 50 mg/kg bw/d in one study. The reliability of NOAEL data was also low. Researchers were unable to determine NOAEL values in 6 of 9 reports due to inadequate dose level design.
- Renal and hepatic injury were the most frequent findings, and evidence suggests unborn rats can acquire the same pathologies when pregnant rats are dosed with MO seed products. Pregnant and breastfeeding women have been cautioned against the consumption of MO seed products as a result of these findings.
- Evidence of renal injury was commonly limited to histopathology, with only one instance of supporting biochemical evidence. Renal injury was not detected in two sub-acute rodent studies, and three sub-acute rodent studies did not include tests for renal histopathology nor renal biochemistry. Due to conflicting evidence, limited sample size and low research quality, it was not possible to determine whether MO seeds are a cause of AKI.
- Isothiocyanates, nitriles and lipids are theoretical toxic fractions of MO seeds. Lipids can be extracted with non-polar solvents, such as n-hexane; isothiocyanate and nitrile production can be occluded during MO seed processing by heat-inactivating myrosinase prior to the addition of water.
- Heat-inactivation of MO-derived myrosinase is theorised to occur at ≥ 40°C (313.15 K). MOCPs are reportedly heat-stable to ≥ 90°C (363.15 K), so this process should not interfere with coagulation potential in water treatment. No additional phytochemicals were identified that undergo thermal decomposition at 40–90°C (313.15–363.15 K). This theory requires additional research.
- MO seed consumption may begin to cause toxicity at 3.1 g/d (~10 seeds per day) in adult humans (mean body mass: 62 kg). Edible nuts are generally portioned at 30 g/d, so it is unlikely that MO seeds can be incorporated into the human diet without risking toxicity via overconsumption.
- Barring consistent failure of CFS or consistent coagulant overdosing, MO seed-treated drinking water is unlikely to cause toxicity as a lone source of phytochemicals. However, dietary and medicinal uses of MO must be considered to avoid toxicity via compounding exposure.
- Allergenic MO seed proteins are an overlooked disadvantage of MO seed water treatment. Reports of allergy are emerging as MO usage increases, though the true scale of the problem is difficult to discern. Five of the “big nine” food allergens are plant seeds (peanuts, tree nuts, soya, sesame and wheat grain), so special attention must be paid to MO seed products in context of allergy.

### 4.1. Recommendations

- Researchers should report MO seed age and storage conditions alongside source location and species verification data. Phytochemical profiles may change as MO seeds age under varying conditions.
- MO seed processing methods require careful consideration, full explanation of reasoning and thorough data reporting. Complexity of processing methods (primary, secondary or tertiary), choice of solvent (e.g., water, ethanol, methanol) and exposure to water and/or elevated temperature (>30°C / 303.15 K) can affect the toxicity of MO seed products.
- Adhere to OECD guidelines, or comparable contemporary guidelines, when designing acute and sub-acute oral toxicity tests [101–103]. Consider the default OECD dosing scheme for acute studies: level 1: 1.75 mg/kg bw; level 2: 5.5 mg/kg bw; level 3: 17.5 mg/kg bw; level 4: 55 mg/kg bw; level 5: 175 mg/kg bw; level 6: 550 mg/kg bw; level 7: 1750 mg/kg bw; level 8: 5000 mg/kg bw [101]. In lieu of an OECD recommendation, consider the following four-level dosing scheme for sub-acute studies: level 1: 30 mg/kg bw/d; level 2: 100 mg/kg bw/d; level 3: 300 mg/kg bw/d; level 4: 1000 mg/kg bw/d; this scheme has an approximate dose progression factor of 3 and theoretically progresses from no toxicity to non-fatal toxicity in rodents dosed with MO seed.
- Include full biochemical tests for renal and hepatic function and injury, just prior to (or during) animal sacrifice. OECD recommend biochemical tests for serum sodium, potassium, glucose, total cholesterol, urea, creatinine, total protein, total albumin, ALP, ALT, AST and bile acids [102]. Compliment biochemical findings with histopathological tests on all preserved organs and tissues, with particular emphasis on the kidney and liver. These finding can be used to assess the involvement of MO seed consumption on AKI epidemiology.
- To test the theory of myrosinase heat-inactivation, toxicology researchers can consider the following experiment: bake whole MO seeds at temperature intervals (e.g., 20°C, 40°C, 60°C, 80°C, 100°C, 120°C, 140°C / 293.15 K, 313.15 K, 333.15 K, 353.15 K, 373.5 K, 393.15 K and 413.15 K) prior to processing and conduct toxicology tests at each temperature interval. Consider *in vitro* exploratory tests (e.g., human cell lines, organoids, organ-on-a-chip), to determine an approximate inactivation temperature for subsequent rodent studies. Exploratory tests protect animal welfare by refining experimental design prior to animal testing.
- Protect animal welfare by adhering to the recommended maximum dosing concentrations: 5000 mg/kg bw for acute studies and 1000 mg/kg bw/d for sub-acute studies.
- Cease promoting MO seed safety and consumption until NOAEL and LD50 values are reliably defined. Current values for oral administration are derived from small sample sizes (n = 2 for LD50, n = 3 for NOAEL) and unreliable studies. If rodent testing guidelines cannot be followed with reasonable fidelity, protect animal welfare by conducting *in vitro* testing (e.g., human cell lines, organoids, organ-on-a-chip).

## Supporting information

S1 Fig Flow chart used to make judgements in bias domain 1: risk of bias due to experimental design and confounding factors (B-J1). (TIF)

S2 Fig Flow chart used to make judgements in bias domain 2: risk of bias due to missing data (B-J2). (TIF)

S3 Fig Flow chart used to make judgements in bias domain 3: risk of bias due to selective reporting of data (B-J3). (TIF)

S4 Fig PRISMA 2020 abstract checklist for data reporting in systematic reviews [129]. (TIF)

S5 Fig PRISMA 2020 manuscript checklist for data reporting in systematic reviews [129]. (TIF)
